# A multi-scale lifecycle and technoeconomic framework for higher education fleet electrification

**DOI:** 10.1038/s41598-024-54752-z

**Published:** 2024-02-28

**Authors:** Jason Juang, Wyatt Green Williams, Arjun T. Ramshankar, John Schmidt, Kendrick Xuan, Joe F. Bozeman

**Affiliations:** 1 College of Business, Georgia Institute of Technology, Atlanta, GA 30322 USA; 2 Georgia Institute of Technology, Civil and Environmental Engineering, Atlanta, GA 30322 USA; 3 Computer Science, Georgia Institute of Technology, Atlanta, GA 30322 USA; 4 Mechanical Engineering, Georgia Institute of Technology, Atlanta, GA 30322 USA; 5 School of Public Policy, Georgia Institute of Technology, Atlanta, GA 30322 USA

**Keywords:** Fleet electrification, Higher education, Electric vehicle, Life cycle assessment, Techno-economic analysis, Carbon payback, Sustainability, Drawdown, Environmental impact, Energy infrastructure, Civil engineering, Energy and behaviour, Energy management

## Abstract

Transportation accounts for one-quarter of all energy related greenhouse gas emissions. As it pertains to transport electrification, higher education institutions—such as universities—can model solutions that affect broader society. Despite this, higher education’s role in fleet electrification adoption has been understudied. We, therefore, modeled an archetypical higher education institution to analyze the carbon and economic payback periods of three electrification scenarios (Business-as-Usual, Targeted Electrification, and Full Electrification) using a cradle-to-grave lifecycle and technoeconomic approach. Given the archetypical higher education institution fleet of 368 vehicles, results show an economic ratio plateau point of about 8 years at 20 fuel-based cars replaced by electric vehicles and a carbon payback period peak of roughly 10 months at 50 fuel-based cars replaced. We then performed a multi-scalar analysis by leveraging implementation theory. We find that higher education institutions that adhere to the tenets of implementation theory are poised to be pro-environmental change agents in many regions and countries. The methods and findings herein can be adapted to other institutions, regardless of fleet size, and can bolster relevant decision-making outcomes now.

## Introduction

Transportation accounts for one-quarter of all energy-related greenhouse gas (GHG) emissions and is also one of the largest sources of urban and regional air pollution due to the combustion of fossil fuels^1^. As it pertains to transport electrification, higher education institutions—such as colleges or universities—can model solutions that are applicable to broader society. Higher Education has a strong market presence of over 25,000 universities worldwide. These institutions have the potential to play an important and transformative role in global sustainability, although higher education itself accounts for less than 2.5% of global energy use and GHG emissions^2,3^. For instance, higher education institutions have made commitments to sustainability and fleet electrification^4^, empowering other organizations to do the same and can play an influential role in local communities as agents of sustainable development^5^. The present study provides meaningful data and a methodological blueprint for other higher education institutions, nationally and internationally, to analyze their sustainability efforts and to serve as regional change agents.

Many higher education institutions have begun to evaluate the electrification implications and needs of their own transport fleet. For example, a study at the University of Dayton in the U.S. investigated the cost effectiveness of a fully electrified, renewably powered and carbon–neutral campus by year 2025^6^. Other universities have set sustainability and net-zero goals but have not applied evaluations to fleet operations. This gap provides a timely opportunity to test the feasibility of developing a roadmap toward net-zero fleets and to garner regional adoption of fleet electrification^6,7^. The present study helps to fill this gap.

Fleet electrification research and development that do not have a higher-education focus (e.g., municipal, commercial, and federal fleets) have had more robust analyses to date compared to higher education institutions. These include analyses on the environmental and social benefits of electric vehicles (EVs), cost perspectives, EV social acceptance, and implementation feasibility^8–11^. There are also relevant examples at the municipal, corporate, and commercial level^10–12^. We highlight one at the municipal level given its methodological relevance. The city of Houston, Texas of the US joined the Energy Secure Cities Coalitions—including the city of Atlanta, Georgia—to evaluate their potential for carbon reduction in hybrid and battery EVs using the Greenhouse Gases, Regulated Emissions, and Energy use in Transportation (GREET) model^13^.

Life Cycle Assessment (LCA) on EVs has improved over time to include more steps and processes specific to the production, distribution, use-phase, and end-of-life processes^14,15^. These improvements include transparency around EV production, appropriate electricity grid mix assumptions, and specific EV comparisons (e.g., a cradle-to-grave comparison of a Ford Transit Van against a Ford E-Transit Van)^16,17^. The use phase and electricity mix of the electricity grid in EV LCAs have been highlighted as two important factors in evaluating EVs in comparison to other transport technology^18^. Previous LCA research has analyzed the future projection of electricity mix scenarios in different countries or regions, such as Lithuania or Hong Kong, and their grid's forecasted increase in renewable energy adoption^19,20^. Future international grid projections show subsequent carbon benefits for EV development. Additionally, LCA research for EVs has expanded to include electricity production, battery recycling, and battery efficiency fade, which helps address fleet electrification uncertainty^21^.

Given these findings, we use the Georgia Institute of Technology (Georgia Tech)—a prominent university located in the southeastern region of the U.S.—as the archetypical higher education institution for other similarly dispositioned institutions and as a testbed for fleet electrification and implementation theory in the present study. Georgia Tech has set targets of reducing its GHG emissions by year 2030 and achieving carbon neutrality by year 2050 with specific strategies to reduce its fleet GHG emissions^22^. The main goal of the present study is to provide a lifecycle framework for higher education institutions to internally adopt and regionally influence transport fleet electrification. We perform three objectives to meet this goal: (1) Analyze Georgia Tech’s fleet through LCA and technoeconomic analysis; (2) Develop a Targeted Electrification framework for high impact carbon reduction; and (3) Investigate the national and international socioecological implications of higher education fleet electrification.

## Results

The results are provided along a spatial scale continuum, from the local scale—our archetypical higher education institution—to the global scale—national and international implications. That is, we begin by showing results for Georgia Tech across each scenario (i.e., Business-as-Usual, Targeted Electrification, and Full Electrification) and provide analysis on the technoeconomic effects of the Targeted Electrification scenario. Then, we move to results for the national and international implications of higher-education fleet electrification using the same Targeted Electrification scenario. This analysis investigates the energy mix of electricity grids relative to our archetypical institution data and assumptions. We also discuss health equity, bottom-up research implications, study limitations, and future directions for the present study.

### Institutional scenario impact analysis

Between the study period of August of 2021 and 2022 for the archetypical institution, the total embodied GHG emissions is 1113 MT CO_2_e when assessing the cradle-to-grave lifecycle of the existing 368-vehicle fleet (i.e., the Business-as-Usual scenario). Of this total, the use-phase GHG emissions amounts to 1036 MT CO_2_e. This translates to the use-phase emissions accounting for roughly 93% of total lifecycle emissions for the internal combustion engine vehicles (ICEVs).

Comparing the Business-as-Usual to the Full Electrification scenario, where all ICEVs are replaced by a suitable EV, the total embodied emissions drop to 274 MT CO_2_e (a 75% decrease), while the use-phase emissions drop to 139 MT CO_2_e (an 87% decrease). The use-phase emissions account for roughly 51% of the total lifecycle emissions in the Full Electrification case. Overall, this scenario yields a carbon savings of 839 MT CO_2_e.

It was imperative to evaluate the distribution of the carbon emissions based on the usage of the fleet keenly, given that the use-phase emissions of the Business-as-Usual scenario accounted for over 90% of overall lifecycle GHG emissions. To do so, we evaluated the Targeted Electrification scenario systematically. The Targeted Electrification scenario ‘targets’ 50 of the most emissive ICEVs for EV replacement. These 50 ICEVs account for 50% of the total GHG emissions of the existing fleet. Our results suggest that ‘targeted’ electrification can yield significant reduction in fleet GHG emissions by replacing only a fraction of the overall fleet (14%). In other words, the overall fleet emissions can be reduced by half with strategic and informed decision making.

### Institutional carbon payback and economic impact results

The existing fleet of the archetypical institution shows a wide range in carbon payback results, from 3 to 1000 months (roughly 83 years). This is due to the varied usage of ICEVs and EVs throughout the year. The inconsistent usage of vehicles skews the carbon payback period range toward 0 months, and thereby reinforces the use of strategic and informed decision-making strategies aimed at replacing the most used and emissive vehicles. However, there are challenges in developing informed strategies in this regard. To facilitate smart higher education fleet investment, administration, and future research, we dig deeper by explaining the economic impacts of the Full Electrification and Targeted Electrification scenario results.

In the Full Electrification scenario, the average levelized cost of an EV was approximately $56,500 USD at a 10% discount rate and $57,000 USD at a 5% discount rate. In the 10%-discount case, this relatively small difference in the levelized costs represents, in part, a lower annual fee for maintenance and electricity. The economic payback period is about 60 years when fully electrifying the fleet.

Looking at the Targeted Electrification scenario, the average levelized cost of an EV was approximately $52,800 at a 10% discount rate and $55,000 at a 5% discount rate. The economic payback period for replacing the top 50 ICEV emitters was roughly 14 years (46 years less than the payback value of the Full Electrification scenario). Interestingly, the entire cost of buying the EVs can be economically recuperated before the end-of-life of modern EVs which is about 15 years—a conservative estimate^23^. This makes a strong economic case for using a ‘targeted’ approach for fleet electrification.

**Figure 1:**
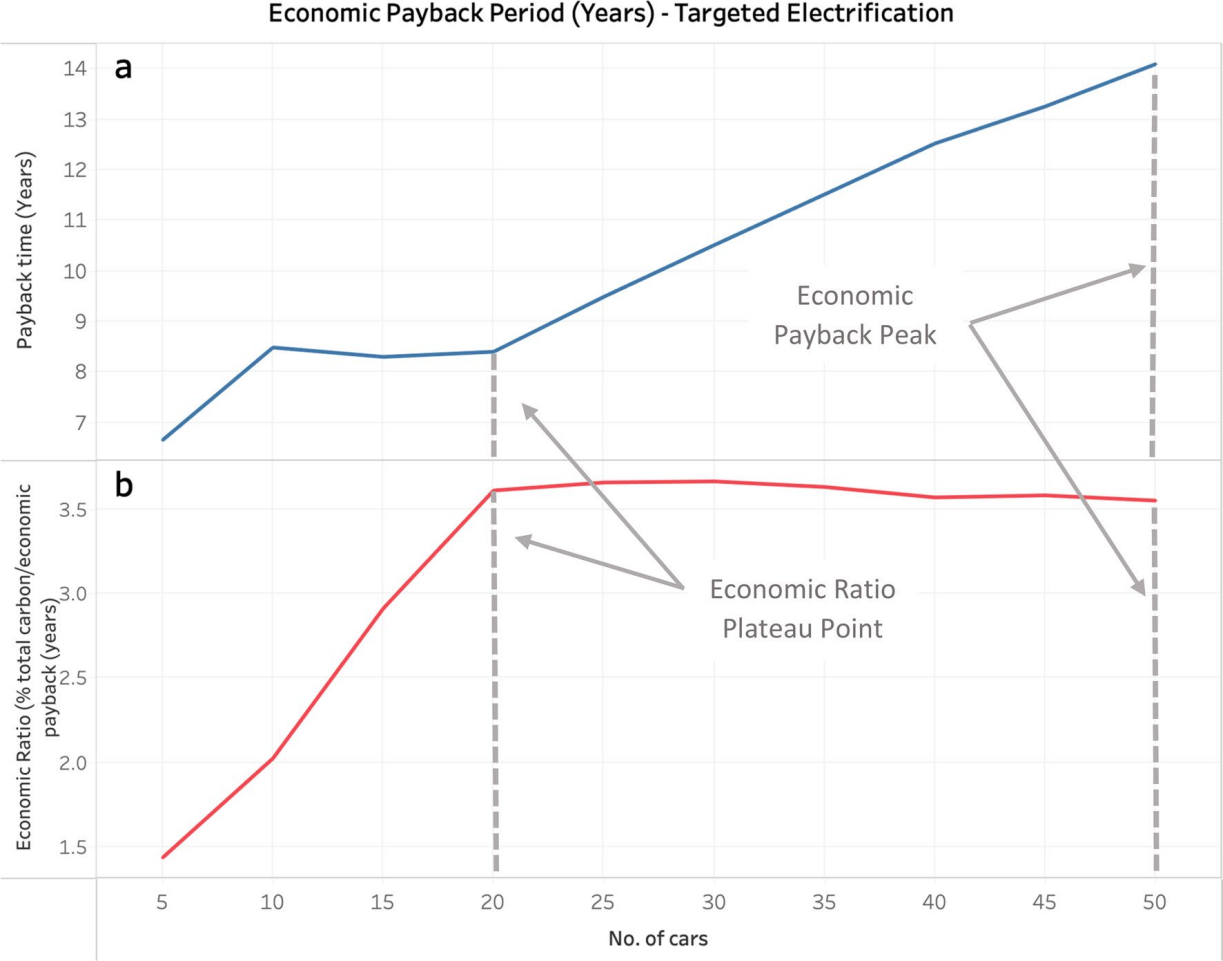
Economic Payback Period (**a**) and Economic Ratio (**b**) for the Targeted Electrification Scenario of the Higher-Education Archetype.

The top portion Fig. 1 (Fig. 1a) shows the trend of the economic payback, and the bottom portion (Fig. 1b) shows the trend of an associated economic (i.e., the percentage of total fleet carbon offset divided by the economic payback time in years) for the Targeted Electrification case. Figure 1a shows that within the 50 highest used vehicles, there is a plateau in the economic payback period from replacing the first 10–20 cars and then a steady increase until all 50 cars are replaced. The curve in Fig. 1b suggests that electrifying the 20 highest used vehicles offsets 30% of fleet emissions while achieving the highest rate of economic payback.

**Figure 2:**
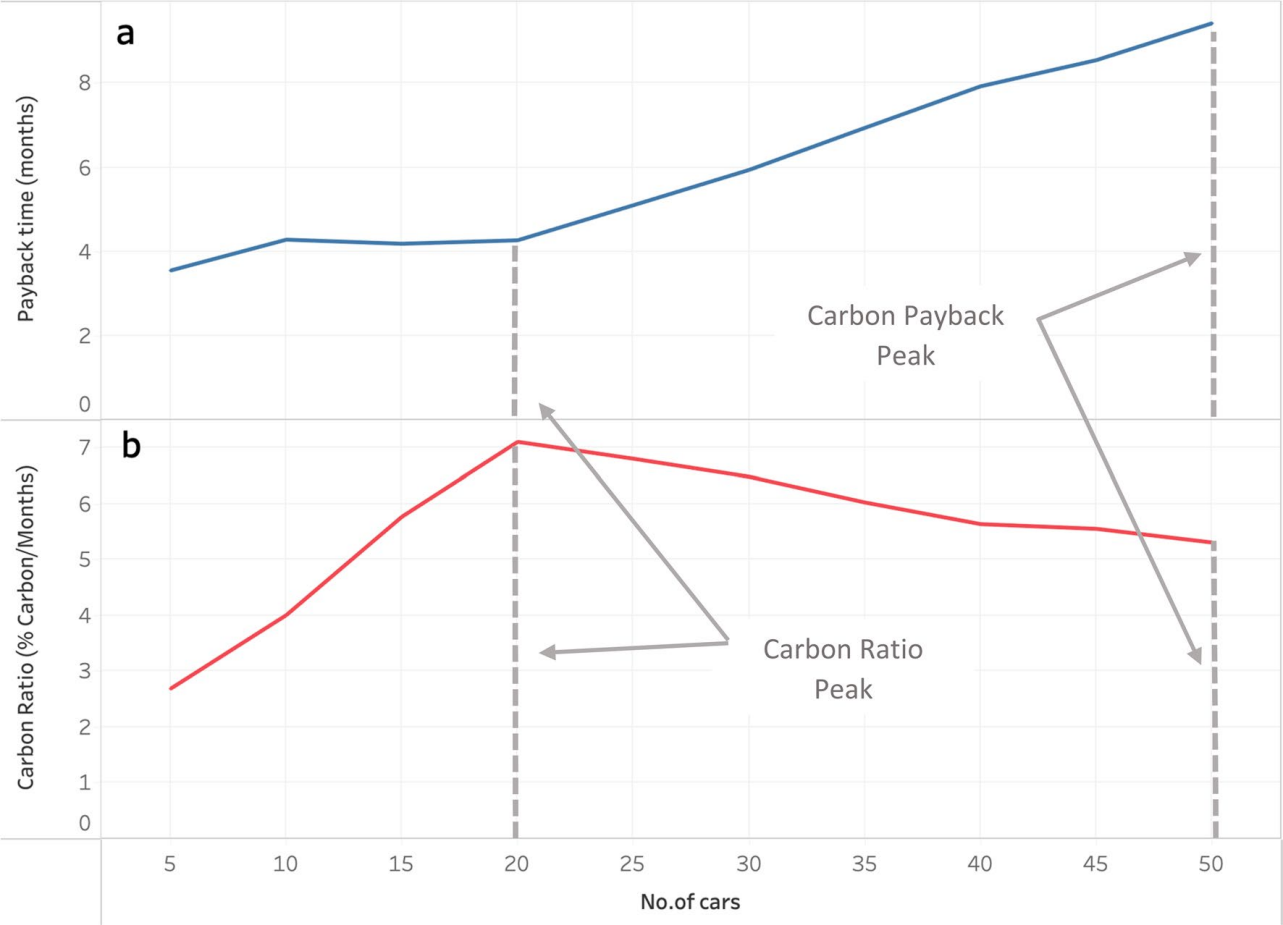
Carbon payback period (**a**) and Carbon Ratio (**b**) for the Targeted Electrification Scenario of the Higher-Education Archetype.

Figure 2a (the top portion of Fig. 2) shows the trend in the carbon payback period for the Targeted Electrification scenario with a payback period ranging from roughly 4 months at 5 vehicles replaced with EVs to 9 months at 50 vehicles replaced. Figure 2b shows that the ratio increases linearly when 5 to 20 vehicles are replaced, where it reaches a peak. This aligns with the archetypical higher education institution results, because replacing the top 20 emitters of the current fleet with EVs provides the highest rate of carbon payback (roughly 4 months) while offsetting 30% of the overall emissions of the fleet. What differs between this and the economic ratio curve is that the carbon ratio dips, rather than plateaus, after its peak at 20 cars replaced. This dip results from a decrease in vehicle usage relative to the top 20 targeted vehicles. On the other hand, the economic ratio (Fig. 1b.) plateaus because the economic payback period increases proportionally to the total embodied and use phase carbon beyond the top 20 cars in the Targeted Electrification scenario.

### Replacing the top-20 ICEVs for maximum economic benefit

Given that electrifying the 20 highest used vehicles offsets 30% of fleet emissions while achieving the highest rate of economic payback, we focus further on these top-20 emitters. The archetypical vehicle types are the sedan, large sedan, van, and truck. Some higher education institutions may not have the financial capabilities to purchase EVs at scale. Therefore, by focusing on vehicle usage and then vehicle type, the maximum carbon reductions can be achieved with less capital resources.

**Figure 3:**
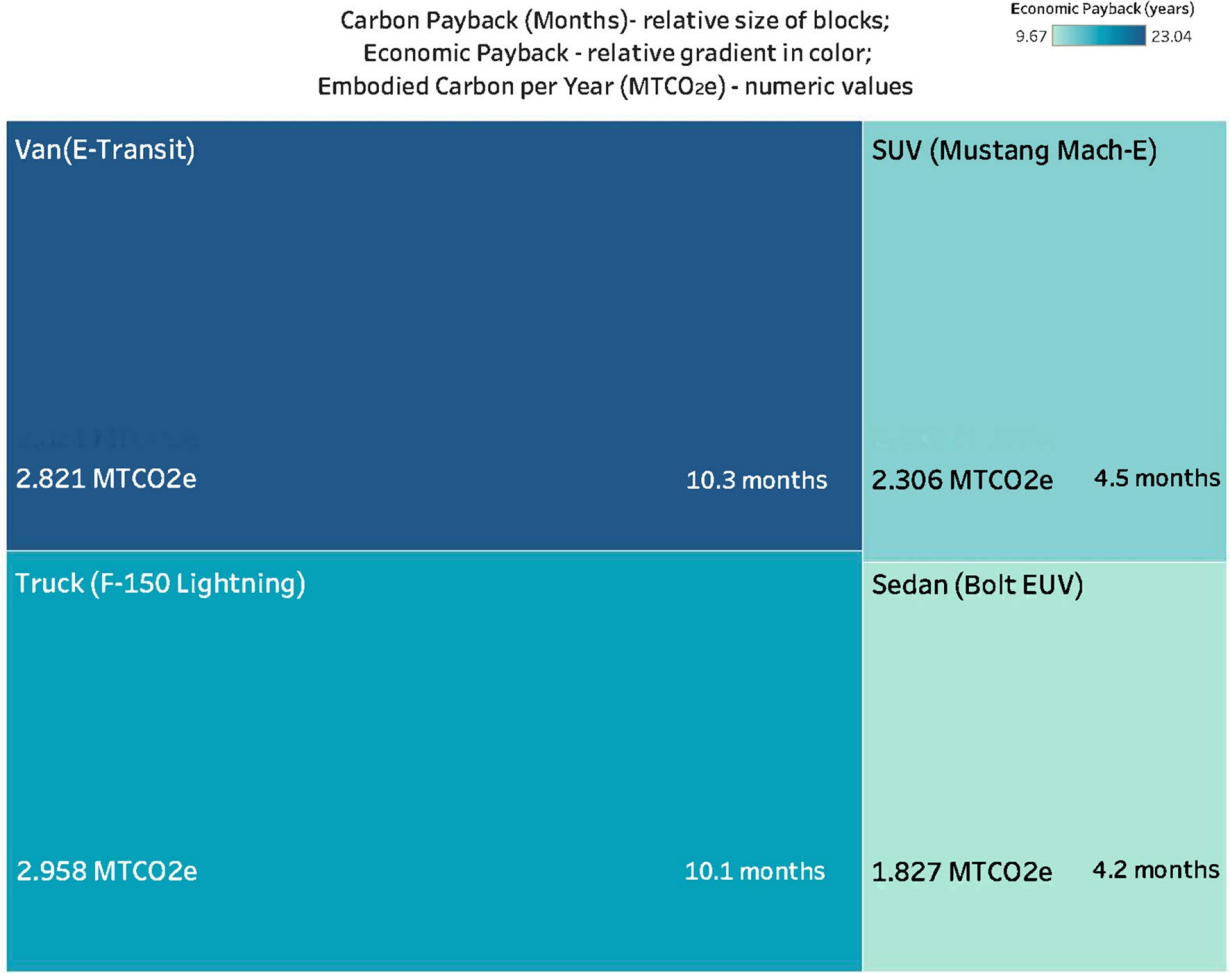
Carbon and Economic Payback Based on Archetypical Vehicle Type.

Figure 3 shows the different carbon and economic paybacks based on vehicle type. The size of the blocks represent the carbon payback achieved, where smaller-sized blocks indicate lower carbon payback time, and larger-sized blocks indicate higher carbon payback time. The color depth represents economic payback, where the lighter color indicates fewer years in comparison to the darker color. It shows that the sedan (Chevrolet Bolt) has the shortest carbon emission reduction and economic payback period, whereas the E-Transit van has the longest. The numbers in each box represent the embodied GHG emissions in MT CO_2_e for each car type over a year and the carbon payback period for each car type. These results suggest that higher education institutions, which do not have detailed vehicle usage data available internally, can realize an effective carbon payback period by electrifying their sedans first.

### Proliferating fleet electrification through implementation theory

Higher education institutions can proliferate regional fleet electrification through implementation theory. Implementation theory is the analysis of how learning processes and their outcomes impact other actors that are affected by the learning process’ topic^24^. Inspired by the pro-environmental policy adoption spillover effects of adaptive capacity theory^25^, we highlight implementation theory to explain how higher-education fleet electrification activities can propel relevant local activities toward regional fleet electrification and beyond. This is done by adhering to a mutual and collaborative set of values in sustainability and electrification outcomes.

For implementation theory to be effective in the context of higher-education fleet electrification, three primary factors should be addressed. First, the higher education institution must have the kind of administrative stature and support for sustainability initiatives that can positively influence other regional actors to electrify their fleets. That is, for a higher-education administration to realize implementation theory in this regard it must have meaningful ties to regional stakeholders and unequivocally champion fleet electrification. Afterall, higher education institutions are significant societal actors that shape local, regional, and national sustainability futures^26^. Second, the higher education institution must collaborate with stakeholder groups of regional influence to develop multi-actor environmental governance. Finally, regional actors must develop intrinsic goals, methods, and support for their own fleet electrification, using the archetypical fleet electrification pathway as inspiration or guidance. When these three factors are addressed, regional actors can realize their goals of fleet electrification through implementation theory by learning from a neighboring institution that underwent or is undergoing a similar transformation.

Once the implementation theory factors have been addressed, the archetypical institution can apply insights for regional fleet electrification through co-research and by leveraging expert opinion. Co-research is a collaborative research approach that involves active participation and cooperation between multiple stakeholders, including the academic researchers, administration from the archetypical institution, and the local actors involved. Expert opinion, in this context, represents insights, knowledge, and guidance that institutions or individuals (e.g., private industry, consultants, engaged community members, or community-based organizations) can offer to planning and implementation, thereby influencing regional action or beyond.

### Electricity mix analysis for the US

The use phase of EVs accounts for more than half of its overall lifecycle emissions. This means that the carbon footprint of an EV over its lifecycle depends heavily on the carbon intensity of the electric grid from which it is powered. This led us to analyze the impacts of fleet electrification based on each U.S. state’s electric grid profile, as it provides useful guidance for higher education institutions. We find that states with more carbon intensive electric grids are unable to achieve the lowest carbon pay back periods from fleet electrification. Our comparison reveals which states can benefit most from fleet electrification, while also illuminating which states need to ramp up grid decarbonization efforts before being able to realize the full benefits of vehicle electrification.

To provide a feasible illustration of the U.S. electricity mix implications for fleet electrification, we used the Targeted Electrification scenario. When we say feasible here, we mean that we chose the scenario that is more likely to be implemented by higher education institutions that electrify their fleet in the near term (e.g., within the next 10 years or so).

**Figure 4:**
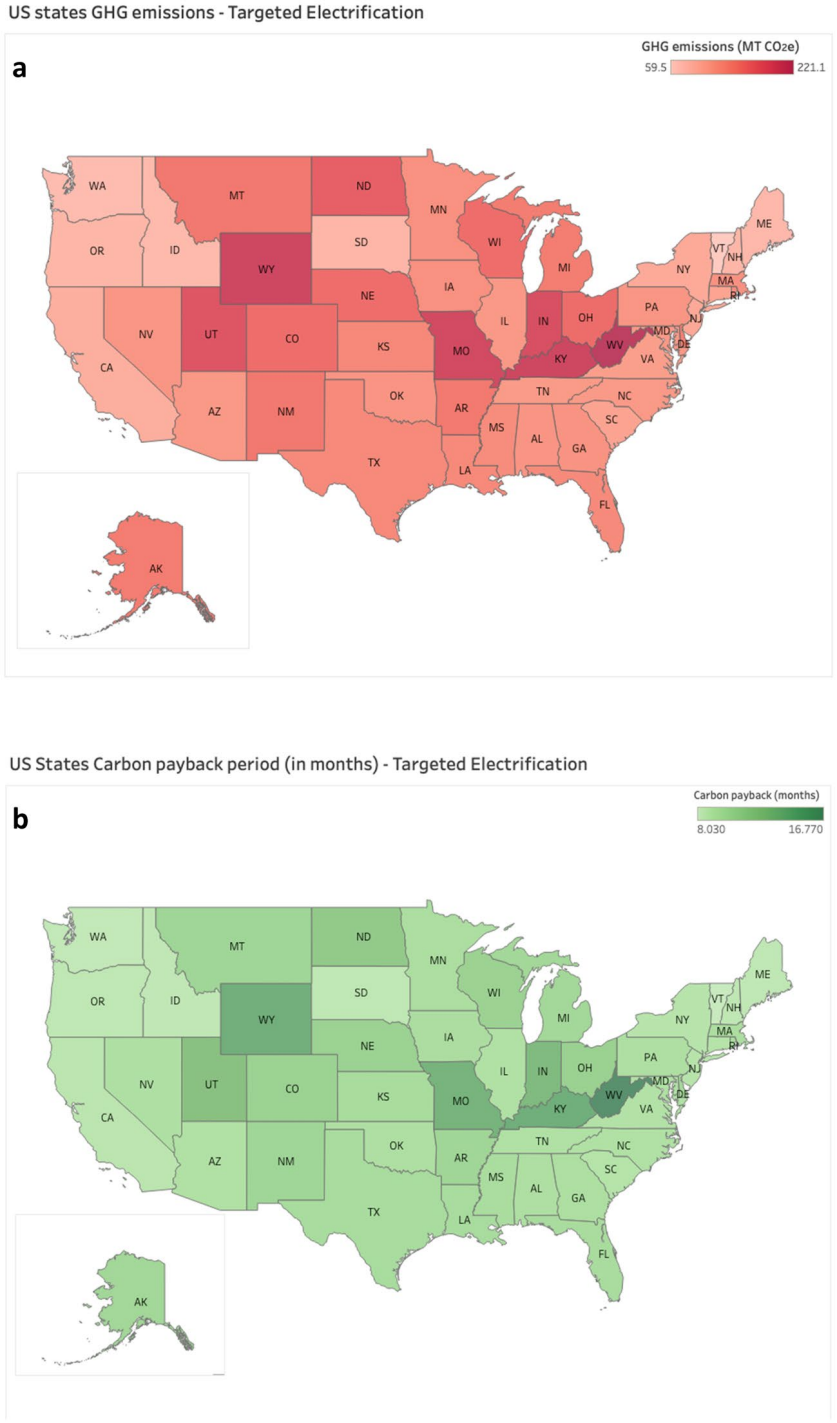
State-wide GHG Emissions (**a**—the top portion) and Carbon Payback Period (**b** – the bottom portion) for the Targeted Electrification Scenario across the U.S. (Note: Tableau Desktop Professional Edition—2023.1.3; https://www.tableau.com/legal/eula).

Figure 4a displays the cumulative GHG emissions for the Targeted Electrification scenario of each U.S. state. Please note that cumulative GHG emissions refers to the embodied and operational net emissions over one year. Figure 4b illustrates the carbon payback period using Georgia Tech’s fleet usage data within the carbon emissions of each state’s electricity grid mix.

There are interesting findings that are worth highlighting here. Vermont, which has a 99% renewable electric grid^27^, has the lowest emissions in the U.S. (59.5 MT CO_2_e). This is almost four times less than that of West Virginia, which has the highest (222 MT CO_2_e) (refer to Fig. 4a). Figure 4b shows the carbon payback period for the Targeted Electrification scenario. The carbon paybacks range from 8 to 17 months, which means that replacing the 50 most used vehicles with EVs would offset carbon emissions within 8–17 months of operation time anywhere in the U.S. In other words, the highest carbon emitting grid in the US only has a 17-month carbon payback period. Fleet EV operation beyond the payback period would result in carbon savings when compared to the Business-as-Usual scenario. These results suggest that higher-education fleet electrification under the Targeted Electrification scenario can play a significant role in decarbonizing the U.S. transport sector.

### International fleet electrification

**Figure 5:**
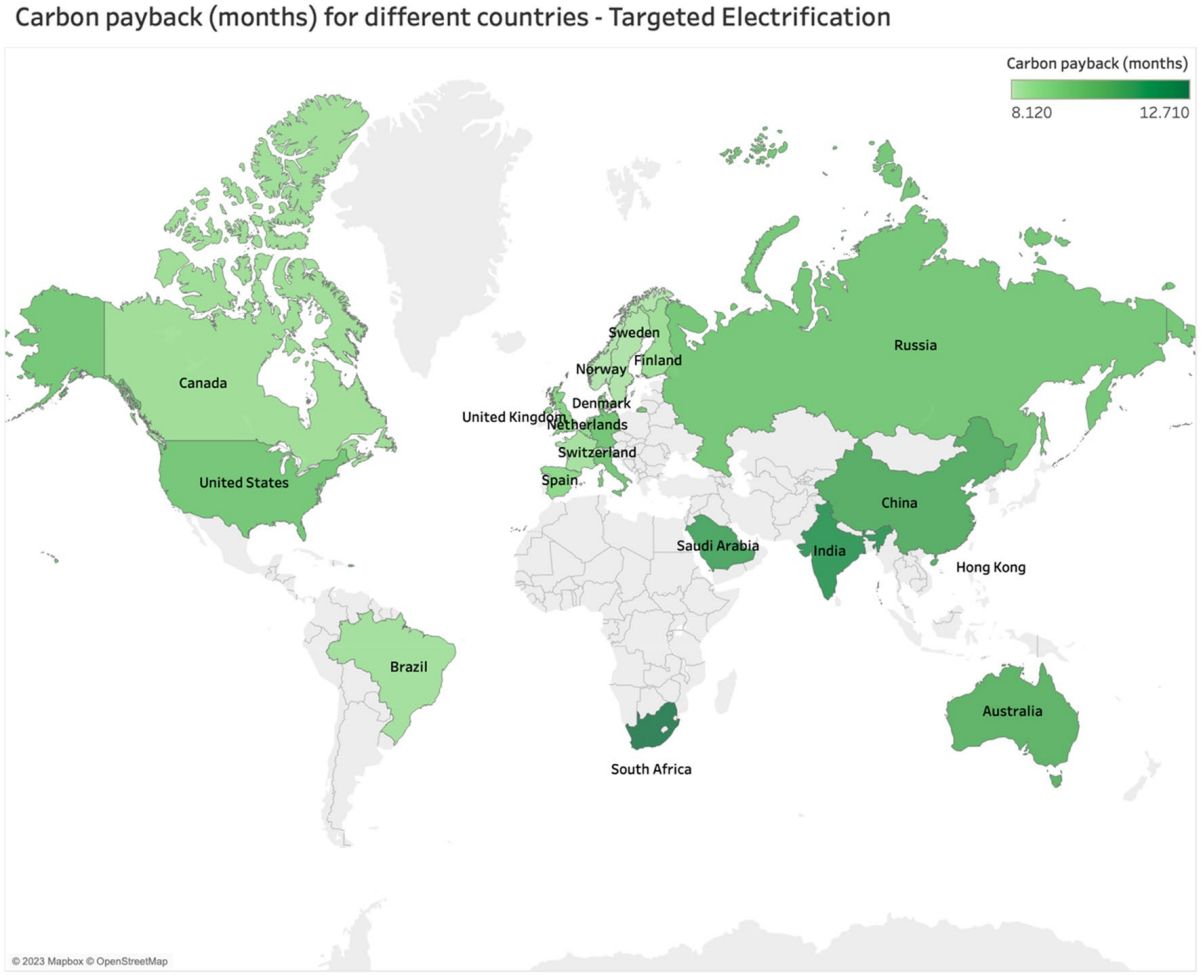
Global Carbon Payback Period for the Targeted Electrification Scenario (Note: Tableau Desktop Professional Edition—2023.1.3; https://www.tableau.com/legal/eula).

Leveraging implementation theory allows us to explore international implications. Again, using the Targeted Electrification scenario, we find that higher education institutions outside of the U.S. can also realize carbon reduction benefits by electrifying their fleets regardless of electricity mix, as the carbon payback periods are lower than 12 months (see Fig. 5). The countries shown in this figure were chosen as a representative sample of the range of global electricity mixes. Figure 5 illustrates the carbon payback period using Georgia Tech’s fleet usage data within the carbon emissions of each country’s electricity grid mix.

Countries with higher education institutions that do not immediately benefit from the Targeted Electrification scenario include Hong Kong and South Africa. This is due to their high carbon grid intensities. While the country level analysis provides an idea of which nations can benefit the most from fleet electrification (see Fig. 4), further granularity is required for realizing optimal carbon payback periods. In comparing two countries—the US and India, the US broadly has a lower carbon payback period compared to India, however, Fig. 4b reveals that certain states within the US exhibit a higher level of carbon emissions when compared to the composite grid mix of India. This implies a need for considering the range of grid mixes within a country before adopting full fleet electrification, given that a nation's average grid emission factor could imply a lower carbon payback period but regionally have a more carbon-intensive effect. Furthermore, at the international scale, the results show that electrification of high-use fleet vehicles results in a favorable carbon payback period. Taken together, fleet electrification has high potential for replicability in international, higher-education settings and helps to address the global challenge of transitioning to EVs^6^.

## Discussion

In meeting our three study objectives, the results reveal fleet electrification strategies that are GHG lowering and economical. A major finding in achieving Objective (1) and (2) was that 50 out of a 386-vehicle fleet—for our archetypical higher education institution—accounted for 50% of the total fleet lifecycle emission. Our findings also suggest that first replacing sedan ICEVs with their EV alternative(s) provide the best combination of carbon emission reduction and payback outcomes for institutions that are constrained in employing full electrification or lack detailed internal data on fleet use. Higher education is often framed as sources of research output, but the present study posits that these institutions can also be important influencers in widespread fleet electrification through implementation theory.

### Health and equity considerations

Regardless of where in the world these fleet emissions emerge, tail pipe emissions, carbon dioxide, nitrous oxide, and particulate matter from ICEVs are a major source of pollution and are detrimental to the health of communities. The exposure to particulate matter and vehicular emissions are connected to cardiovascular morbidity, mortality, and cancer which endangers the students, faculty, employees, and surrounding community of higher education institutions^28,29^. The corresponding social effects, ranging from educational attainment to household income, are correlated with these adverse human health outcomes. Furthermore, lower-income and systematically marginalized communities are too often disproportionately burdened with these inequities^30–32^.

The electrification of vehicles represents an important component of a holistic approach to mitigating adverse health and equity outcomes. For example, the U.S. is forecasted to prevent an estimated $17 billion USD in annual health and climate mitigation costs by implementing a 25% increase in EV adoption at the national level^33^. To help realize these health and climate mitigation benefits in marginalized communities, it will require, in part, the urging of policymakers to administer equitable laws and programming for daytime EV charging and baseline energy demand reductions^34^.

### Empowering bottom-up programming in higher education

The present study exemplifies how collaborative and bottom-up, student-led activities can yield meaningful outcomes in a higher-education context. The data gathering and preliminary analysis work performed by the student-led organization, ElectrifyGT, led to the creation of an internal report on fleet electrification for the archetypical higher education institution (i.e., Georgia Tech). These previous report findings were presented to Georgia Tech administration and led to ElectrifyGT students joining the development of Georgia Tech’s 2022 Climate Action Plan^22^.

There are other ElectrifyGT reports that have had tangible institutional impact. For example, a report presented to the Georgia Tech Police Department resulted in the purchase of three Mustang Mach-Es and two new EV chargers being installed at their building^35^. Another report to Georgia Tech Landscaping Services and Georgia Tech Infrastructure and Sustainability resulted in the electrification of landscaping equipment including leaf blowers, lawn mowers, and chainsaws. These improvements have had benefits in reducing noise pollution reductions, health risks for employees, and improving equipment life span^36^. Support from upper-level, higher-education administrators is vital in empowering bottom-up collaboration and future research.

### Study limitations and future directions

There are some factors that should be highlighted regarding the limitations of the present study and promising future directions for research. The limitations include state contract barriers preventing certain EV alternatives from being considered and constraining energy contract terms between the archetypical higher education institution and its power utility. The future directions largely center on how and which research aspects could be improved to build upon the present study framework.

Vehicles that were not included in the scope of the present study for ICEV replacement encompass heavy duty, all-terrain, stripped chassis, and hybrid vehicles (i.e., vehicles that use carbon-based gasoline as a primary fuel source). The alternative EVs suggested in the present study aligns with the terms of the University System of Georgia Statewide Vehicle Availability Contract, dated June 7, 2022. These terms limit the types of vehicles the archetypical institution can legally purchase. Furthermore, medium duty vehicles such as bucket trucks were not included due to their lack of market-competitive electric alternatives. Additionally, all-terrain vehicles were not included due to their limited fleet presence and usage at the archetypical higher education institution. Hybrid vehicles were not included due to their unclear electrification categorization and relatively low usage at the same institution.

The archetypical institution has a unique relationship with its utility provider for electricity. It allows for the purchase of electricity at a discounted price in comparison to what typical households or even other higher education institutions might pay. These factors contextualize the implementation theory, environmental, and economic impact findings of the present study.

Future research directions largely center around examining how to bolster the analytical capabilities and replicability of the present study framework to other institutions, such as exploring more incremental electrification processes, applications to pertinent non-higher education institutions (e.g., government fleets), and how fleet electrification might overburden electric grids. Future research should also explore the application of the benefits of higher-education fleet electrification in similar institutions (e.g., government and corporate institutions). The fleet analysis framework established by the present study can be adapted to virtually any fleet, regardless of size or institutional type, so long as fleet usage information is tracked in a similar fashion. It is worth noting that fleet characteristics and institutional factors—such as existing fleet size and the number of EVs that may already populate the fleet—will have dynamic effects on carbon and economic payback outcomes.

Future research should also address how other types of higher education institutions may have to overcome distinct barriers to fleet electrification. These include institutions that have historically been marginalized or under resourced such as Tribal Colleges and Universities and Historically Black Colleges and Universities in the US^37^. Depending on the specific characteristics of the institution, these distinct barriers could include overcoming inequitable access to the capital needed to purchase EVs, challenges in electricity rate negotiations with utilities, and barriers to effectuating regional fleet electrification due to these foundational resource issues. The present study framework can only realize its potential for proliferating fleet electrification when the challenges of institutions like these are holistically addressed.

Issues regarding the strategic planning of EV charging periods represent another promising research area. Charging strategies have received some investigation such as in industrial and commercial contexts^11,38,39^. However, we find that there is limited investigation on charging strategies for higher education institutions^40,41^. Further work must be performed to ensure that higher-education policies and guidelines facilitate the social and economic benefits of fleet electrification. Furthermore, as broad investment in electric micro-mobility increases, LCA studies should continue to refine technoeconomic impact analyses, end-of-life management, and circular economy applications for more robust findings and recommendations^42^.

## Methods

LCA allows for the meaningful comparison of two or more technologies or systems on the basis of a relevant functional unit^43^. We have defined our functional unit by segmenting our four archetypical vehicle types: van, truck, SUV, and sedan. The respective functional units for each vehicle type had a profile of an American BEV with an assumed lifespan of 150,000 km^14^. The functional unit replacements were as follows: 2023 Ford E-Transits replaced ICEV-vans; 2023 Ford F-150 Lightnings replaced ICEV-trucks; 2021 Chevrolet Bolt EV replaced ICEV-SUVs; and the 2023 Bolt Electric Utility Vehicle replaced ICEV-sedans. As a key element in the present study, data and results relied on vehicle replacement contingent on the annual use phase. The more typical functional unit “per km traveled” was not chosen.

**Figure 6:**
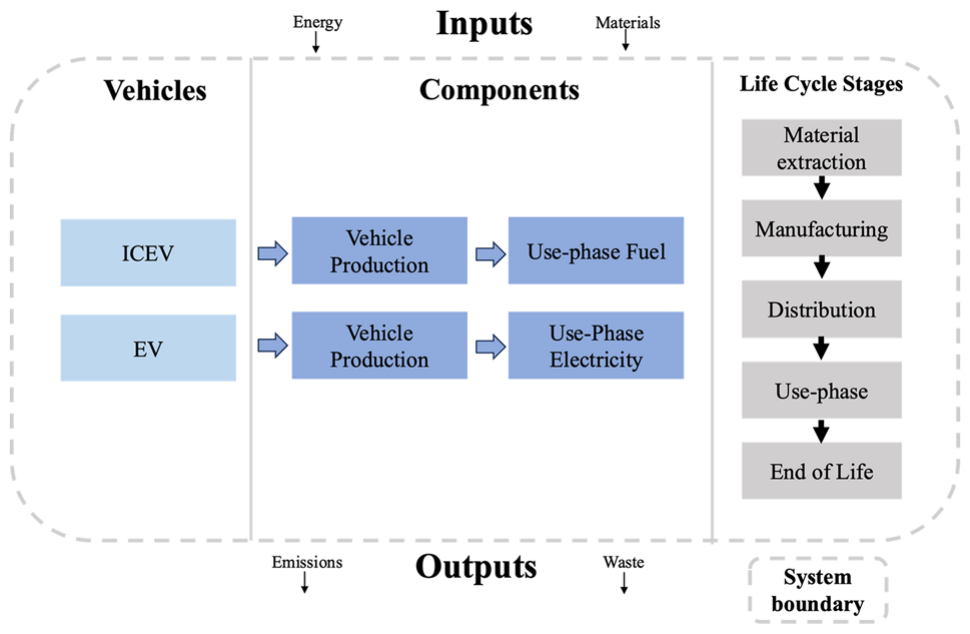
The LCA Stages and System Boundary of the Present Study Cradle-to-Grave Analysis.

In the present study, we used a cradle-to-grave study boundary to model the technoeconomic and environmental impacts of the respective functional unit and their ICEV counterparts. A cradle-to-grave approach estimates the GHG emissions from the sourcing of raw materials—such as the retrieval of metals and elements used in car manufacturing—to the decommissioning of the car at its end-of-life. To achieve this, an inventory of the different emission factors for vehicle specific materials and processes was compiled. This was then translated into quantitative environmental impacts—GHG or carbon emissions—using fleet inventory data, fleet fuel usage, infrastructure costs, vehicular LCA data from GREET^44,45^, and electricity grid emissions from OurWorldinData^46^. Figure 6 shows the primary LCA stages of the present study.

ICEVs and EVs, albeit similar in some ways, have different emission profiles due to material, manufacturing, and disposal distinctions. An example similarity is the glider which represents the vehicle ‘body’. These could be similar in terms of material and supply chain activities. As for an example difference, the vehicle powertrains and source of fuel differ; in that, ICEVs use either diesel or gasoline to operate, whereas EVs effectively use electricity to ‘fuel’ their batteries. These factors affected the development of our LCA model. We provide further detail on this and other key methodological components in the following sub-sections.

### Scenario comparison

As of 2022, the fleet of the archetypical higher education institution (Georgia Tech) consisted of 368 gasoline- and diesel-powered vehicles. We chose three LCA scenarios (i.e., Business-as-Usual, Targeted Electrification, and Full Electrification) that incorporated the replacement of a certain number of these ICEVs with EVs. The present study does not delve into adjusting the overall size of the archetypical institution’s fleet. Instead, we assumed that the overall fleet size was vital to maintain necessary institutional operations.

Each scenario had a unique set of conditions. The Business-as-Usual scenario was where the fleet maintained its current ICEV fleet and did nothing to allocate new EV units. The Targeted Electrification scenario electrified a subset of 50 ICEVs which accounted for 50 percent of the overall fleet's annual GHG emissions. The Full Electrification scenario replaced every ICEV in the fleet with a similarly classed EV in the year following the one-year study period of August of 2021 through August of 2022. While this latter scenario may be unlikely to be realized in a one-year period, this scenario is vital in demonstrating the maximum possible benefit of an electrified fleet and served as a baseline of comparison for the other two scenarios.

### Fleet data aggregation

The present study was performed using data on institutional fleet inventory, power purchasing, infrastructure installation, and vehicle-specific car mileage. Primary data was sourced from the following: Georgia Tech Fleet Management; the Georgia Tech vehicle listing information reported to the Sustainability Tracking, Assessment, & Rating System (STARS); Georgia Tech Utilities; and the Georgia Tech Police Department. Furthermore, vehicle-specific mileage data was gathered from substantiated and publicly available databases^40^.

We now provide specifics on the data gathered. Georgia Tech Fleet Management data included fleet fuel expense and use within the study period of August of 2021 through August of 2022 (Supplementary Data 1.1), the total fuel spend based on type of fuel (Supplementary Data 1.2), an active inventory list of vehicles that were currently in operation at the archetypical institution (Supplementary Data 1.3), a vehicle availability report (Supplementary Data 1.4), and a list describing the types of vehicles that the archetypical institution could purchase as outlined by the applicable University System of Georgia State Contract.

Vehicle listing information was sourced from STARS as a part of the Association for the Advancement of Sustainability in Higher Education (AASHE). This is an organization that aims to improve sustainable practices in higher education institutions through advocacy and data aggregation^47,48^. Information given to AASHE was self-reported by Georgia Tech and was submitted by the Sustainability Program & Portfolio Manager in the Office of Campus Sustainability. This fleet information detailed vehicle classifications based on fuel type, vehicle number, year, make, vehicle identification number, model, department, custodian, and type of vehicle. Fleet management and STARS data was compiled into a comprehensive dataset to provide insight into how the fleet was operated during the reporting period (Supplementary Data 1.5).

There were other data types connected to the primary sources previously mentioned. The Georgia Tech Police Department data provided values on the installation costs of two EV Charge Point chargers. These included financial information on charging stations and electrical run among other relevant fees. The data we collected from the Georgia Tech Utility provided information on hourly real-time pricing, the two-year historical average marginal cost of a kWh, the average cost of a kWh in off-peak hours, the average cost in off-peak hours, and the average cost in on-peak hours. For the sake of replicability, it is worth highlighting that the latter data—between the archetypical institution and the Georgia Tech Utility—is under a non-disclosure agreement.

### Fleet data processing

From the STARS data, fuel use per vehicle was summed to find the total amount of fuel used by the archetypical institution’s fleet. Similarly, fuel and maintenance costs were used to find both the total expenditures of ICEVs and vehicle-specific annual costs. Fuel expenditures and vehicle-specific mileage data were then used to estimate the approximate miles driven by each vehicle during the study. Other data were used to calculate GHG emissions: the fuel use per vehicle in the reporting period, the GHG emission factors of gasoline^49,50^, and the metric tons of CO_2_e emitted per fuel-gallon consumed. To find the total carbon emitted by the fleet, we summed the vehicle-specific emission values.

Each ICEV was matched with an appropriate EV to form the basis of our vehicle-replacement scenario calculations. Each ICEV match was defined with our respective functional unit given their corresponding EV replacement. We used the University System of Georgia State Contracts to assist in this activity.

The miles per gallon equivalent of the EVs and the mileage of the ICEVs were used to find the estimated electricity use for each replacement EV^51^. The replacement EV electricity was summed to find the total electricity of the replacement EV fleet. The replacement EV electricity and the GHG emissions factor of electricity production in the state of Georgia were used to estimate the use-phase carbon emissions of the EV fleet^52^. Furthermore, Georgia Tech utility information was used to find the electricity cost per kWh. Correspondingly, this was included in the cost per EV and the overall replacement costs of the fleet.

The maintenance costs of the ICEVs and EVs were sourced from an Argonne National Laboratory Report and were used in the technoeconomic analysis^53^. The replacement costs for EVs were informed by their corporate websites (Supplementary Data 1.6). The base versions of the cars were chosen and used for analysis. The cost of diesel, gasoline, and electricity consumed were direct costs from an internal source from Georgia Tech Fleet Management and Utilities. The rate pricing information was protected by the non-disclosure agreement previously mentioned. The exact values have, therefore, been excluded from this study to abide by this agreement.

### Principal carbon calculation approaches

Three principal formulas for calculating our key analytical outputs are provided. Embodied carbon refers to the cumulative carbon emissions resulting from various lifecycle stages of the car—such as manufacturing of the components, assembly, lifetime fluids usage, and disposal (see Eq. 1). The carbon payback is defined as the time it would take for the difference in use-phase emissions between an EV and ICEV to surpass the difference in the embodied carbon (see Eq. 2). Equation 3 represents the carbon savings. Calculating this involved integrating aspects of both embodied carbon and carbon payback. It is defined as the net carbon savings per year that are gained from replacing an ICEV with an EV.

1 $${\text{Embodied Carbon }} = \, \left( {{\text{total life cycle carbon}}{-}{\text{use}}{-}{\text{phase carbon emissions per year}}} \right).$$

2 $$\begin{aligned} {\text{Carbon Payback }} & = \left( {{\text{ICEV use}}{-}{\text{phase emissions per year}}{-}{\text{EV use}} {-} {\text{phase emissions}}} \right. \\ & \quad \left. {{\text{per year}})/{\text{ICEV embodied carbon}} {-} {\text{EV embodied carbon }}} \right). \\ \end{aligned}$$

3 $$\begin{aligned} {\text{Carbon Savings }} & = \left( {{\text{ICEV use}} {-} {\text{phase emissions }} + {\text{ embodied carbon }}*{\text{ distance travelled}}/{\text{lifespan of car}}} \right) \\ & \quad {-} \left( {{\text{EV use}} {-} {\text{phase emissions }} + {\text{ embodied carbon }}*{\text{ distance travelled}}/{\text{lifespan of car}}} \right) \\ \end{aligned}$$

### Economic impact approach

Economic investment was calculated by aggregating the net present value of the EVs and the charging infrastructure required to electrify the fleet. The annual costs included maintenance costs and the cost of electricity consumption. Different discount rates were used to arrive at the total cost of ownership of the ‘new’ electric fleet for the Targeted Electrification and Full Electrification LCA scenarios. For clarity, discount rates are a representative percentage of future cash flow that are used to calculate present values^54^. To align with the present study context, it is the investment rate-of-return value for car manufacturers and their associated industry partners. Both scenarios in the targeted and full-electrification scenario used discount rates of 5%, 7%, and 10%.

### Archetypical institution in country selection and electricity mix data

Before we explain the country selection and electricity mix data, it is important to justify the use of Georgia Tech as the archetypical higher education institution through implementation theory. Georgia Tech satisfies the three primary factors of implementation theory, as previously explained (see the *Proliferating Fleet Electrification through Implementation Theory* section), through its administrative support for fleet electrification, leadership in the South East Transportation Regional Initiative (SETRI), and its EV research funding partnerships from regional EV manufacturing actors^55^. In terms of administrative support, Georgia Tech has created a climate action plan, detailing decarbonization policy decisions including the electrification of its fleet vehicles for Scope 1 and 2 emission reduction^22^. As it pertains to multistakeholder collaboration, Georgia Tech leads SETRI—a coalition of automakers, businesses, nonprofits, state agencies, and other entities promoting EV development in the southeast region of the US^56^. Regarding regional actors and their intrinsic EV proliferation goals, Hyundai—a prominent EV manufacturer—has established an EV and battery plant in the region near Georgia Tech and has also formally agreed to collaborate with Georgia Tech in EV research moving forward^57^. This is complemented by new investments from regional EV and cleantech companies such as Rivian and Qcells^58^. This allows the present study to use Georgia Tech as the archetypical higher education institution through implementation theory. It is worth noting that not every institution requires strong partnerships with EV manufacturers and cleantech companies to potentially influence regional fleet electrification using implementation theory. However, research suggests that geographic proximity to immediate neighbors can increase the likelihood of policy adoption^24^. Additionally, any institution that has already electrified can educate and achieve synergy effects with local institutions by sharing fleet electrification information. Institutions that haven’t electrified, or have partially electrified, can use Georgia Tech as an example of collaboration for educational and non-educational institutions to influence their own regional fleet electrification. We argue that the identification of other universities which can use implementation theory and its application to their geographical contexts is important because carbon reduction solutions are most impactful at scale.

Now, we explain the country selection and electricity mix data. Throughout our spatial scale continuum, the fleet use data was kept constant to the varying electric grid mixes of different states and countries to understand the variances of carbon payback periods for the archetypical fleet. Within our comparison of the US and different countries, the fleet size and usage data of our archetypical institution does not change, and the results show only our archetypical institutional fleet electrification represented in different scenarios of electricity mixes. The countries were chosen to represent the range of global electricity mixes- with fossil dominant mixes, such as South Africa, being the most carbon intensive to renewable-dominant ones, such as Sweden, being the least. While we recognize that there are many different types of higher education institutions, we aim to use our archetypical institution as a developing source for future research. The electric grid carbon intensity of different U.S. states and for many countries were collected from the Environmental Protection Agency, OurWorldinData, and Statista^46,49,59^. For clarity, electricity mix is defined as the different sources of primary energy that generate electricity^60^. These grid intensities were assessed as part of the lifecycle in the present study to give the carbon footprint of the higher-education fleet if it were operational in different U.S. states and countries. To yield the associated data visualizations, this electricity mix data was imported into Tableau to make visual maps of the respective grid emissions and payback times.

### Supplementary Information

Supplementary Information.

## Data Availability

All data generated or analyzed during this study are included in this published article [and its supplementary information files].
