# Supplementary material for: A multi-scale lifecycle and technoeconomic framework for higher education fleet electrification
Source: Sci Rep. 2024 Feb 28;14:4938. doi: 10.1038/s41598-024-54752-z (PMC10901860; doi:10.1038/s41598-024-54752-z)
Supplement: Supplementary file 1 — Supplementary Information. [file 41598_2024_54752_MOESM1_ESM.pdf]

A Multi-Scale Lifecycle and Technoeconomic Framework for Higher Education Fleet Electrification

Jason Juang, Wyatt Green Williams, Arjun T. Ramshankar, John Schmidt, Kendrick Xuan, and *Joe F. Bozeman III

Supplementary Information

This file contains a representation of all the data used in the formal analysis of the manuscript. The goal of this file is to present the way in which fleet data is collected in a university setting and the kind of data that is usually tracked and collected.

1.1 Fleet Fuel Expense Tracking:

Unit #	VIN	Vehicle Model	Cost Per Mile	TXN(s)	Units	Spend	Location(s)
503-0198	1FBNE3BL0CDB14274 (Decoded)	2012 Ford Econoline	$0.20	133	2,951.90	$7,431.29	ST OF GA BOR-GEORGIA TECH-Campus
503-1177	1FM5K8AR2JGA46400 (Decoded)	2018 Ford Explorer	$0.41	370	2,381.88	$6,032.08	ST OF GA BOR-GEORGIA TECH-Campus
503-1267	1GNSKLED1MR334207 (Decoded)	2021 Chevrolet Tahoe	$0.28	179	2,027.31	$5,856.37	ST OF GA BOR-GEORGIA TECH-Campus
503-1119	1FM5K8AR2HGB41047 (Decoded)	2017 Ford Explorer	$0.43	252	2,008.32	$5,041.98	ST OF GA BOR-GEORGIA TECH-Campus
503-1264	1FM5K8ABXMGB35039 (Decoded)	2021 Ford Explorer	$0.43	272	1,889.47	$4,895.52	ST OF GA BOR-GEORGIA TECH-Campus
503-1210	1FBZX2YM3JKB37398 (Decoded)	2018 Ford Transit	$0.33	130	1,903.63	$4,830.04	ST OF GA BOR-GEORGIA TECH-Campus
503-1235	1FBZX2YM3KKB13054 (Decoded)	2019 Ford Transit	$0.31	121	1,822.47	$4,574.24	ST OF GA BOR-GEORGIA TECH-Campus
503-1237	1FBZX2YM7KKB13056 (Decoded)	2019 Ford Transit	$0.31	109	1,809.50	$4,555.90	ST OF GA BOR-GEORGIA

							TECH-Campus
503-1236	1FBZX2YM5KKB13055 (Decoded)	2019 Ford Transit	$0.32	110	1,800.87	$4,526.04	ST OF GA BOR-GEORGIA TECH-Campus
503-0786	1FTEW1CWXAFC63248 (Decoded)	2010 Ford F-150	$0.70	118	1,773.92	$4,493.25	ST OF GA BOR-GEORGIA TECH-Campus
503-1263	1FM5K8AB8MGB35038 (Decoded)	2021 Ford Explorer	$0.39	195	1,749.22	$4,438.26	ST OF GA BOR-GEORGIA TECH-Campus
503-1265	1FM5K8AB6MGB35040 (Decoded)	2021 Ford Explorer	$0.43	217	1,738.07	$4,416.83	ST OF GA BOR-GEORGIA TECH-Campus
503-1218	1FM5K8ARXKGA30916 (Decoded)	2019 Ford Explorer	$0.50	193	1,679.56	$4,394.66	ST OF GA BOR-GEORGIA TECH-Campus
503-1194	1GNLCDEC6JR340353 (Decoded)	2018 Chevrolet Tahoe	$0.33	99	1,541.85	$4,390.62	ST OF GA BOR-GEORGIA TECH-Campus
503-0902	2C3CDXAG8EH208950 (Decoded)	2014 Dodge Charger	$0.50	148	1,685.00	$4,193.71	ST OF GA BOR-GEORGIA TECH-Campus
503-1217	1FM5K8AR4KGA53009 (Decoded)	2019 Ford Explorer	$0.44	198	1,638.80	$4,108.55	ST OF GA BOR-GEORGIA TECH-Campus
503-1174	1FM5K8ARXJGA46399 (Decoded)	2018 Ford Explorer	$0.50	182	1,615.00	$4,040.43	ST OF GA BOR-GEORGIA TECH-Campus

503-1024	2C3CDXAG8FH875275 (Decoded)	2015 Dodge Charger	$0.60	126	1,488.77	$3,783.66	ST OF GA BOR-GEORGIA TECH-Campus

1.2 Total Fuel Spend by Type:

Product Type	Spend	Units
Gasoline	$308,068.02	115,900
Diesel	$23,688.05	7,615
Automotive Products/Services	$770.00	1
Other Fuel	$85.74	29
DEF (Diesel exhaust fluid)	$21.90	7
Miscellaneous	$4.21	8

1.3 Inventory of Existing Fleet:

VIN Model	Status	Model Year	Model Name
F-150	Active	2010	F150
IMPALA	Active	2009	IMPALA
WINDSTAR	Active	2002	WINDSTAR
F-250	Active	2005	F250
F-150	Active	2000	F150
F-350	Active	2009	F350
ECONOLINE WAGON	Active	2012	ECONOLINE WAGON
CLUB CAR	Active	2017	CLUB CAR
ESCAPE	Active	2019	ESCAPE
ESCAPE	Active	2019	ESCAPE
L3800FW	Active	2012	L3800FW
ESCAPE	Active	2014	ESCAPE
ESCAPE	Active	2014	ESCAPE
ES	Active	2016	ES
TAHOE	Active	2018	TAHOE

1.4 Vehicle Availability as per State Contract:

Statewide Contract #	Supplier	Class	Vehicle Model	Current Model Year	Base Price	Current Term	ePACT Compliant	Cutoff Dates for Ordering

99999-SPD-ES40199373-004	Griffin Speedway Ford	SUV	Ford Expedition	2022	$40,250.00	Extension	No	No Cutoff date
99999-001-SPD0000183-0004	Family Ford	Electric	Ford Mustang Mach E, RWD	2022	$44,402.00	Initial Term	Yes	No cutoff date established.
99999-SPD-ES40199373-009S	Wade Ford	Passenger Van	Ford Transit T-350 Passenger Wagon, 148 in long WB, 12 Pass., Low Height	2023	$45,483.00	Extension	Yes	TBA
99999-SPD-ES40199373-009S	Wade Ford	Passenger Van	Ford Transit T-350 Passenger Wagon, 148 in long WB, w/ Ext. Body, 15 Pass., High Height	2023	$48,994.00	Extension	Yes	TBA
99999-001-SPD0000183-0002	Hardy Chevrolet	Electric	GM Bolt	2023	TBA	Initial Term	Yes	TBA
99999-001-SPD0000183-0002	Hardy Chevrolet	Electric	GM Bolt (EUV)	2023	TBA	Initial Term	No	TBA
99999-001-SPD0000183-0006	Wade Ford	SUV-PPV	Pursuit Utility (Explorer)All Wheel Drive	2022	**$35.630.00**	Initial Term	No	No cutoff date established.

1.5 Fleet Usage Analysis:

The fleet usage analysis can be accessed using this link: https://docs.google.com/spreadsheets/d/1Zt8r_NYiH6AnBM-pY913klh_ebqXQR-J/edit#gid=852766555

1.6 Vehicle Replacement costs (EVs):

The purchasing cost of each EV was taken from the respective websites:

- 2023 Ford E-TransitTM All-Electric Van

  https://www.ford.com/commercial-trucks/e-transit/

- 2023 Bolt EUV: Electric Utility Vehicle

https://www.chevrolet.com/index/vehicles/2023/suvs/bolt-euv/overview.html

- 2023 Bolt EV: Electric Car

  https://www.chevrolet.com/index/vehicles/2023/suvs/bolt-ev/overview.html

- 2023 Ford® F-150 LightningTM Electric Truck

  https://www.ford.com/trucks/f150/f150-lightning/

- 2023 Ford Mustang Mach-E SUV

  https://www.ford.com/suvs/mach-e/2023/models/

2. Life cycle Analyses of ICEVs and BEVs across different vehicle types

The life cycle data for the different car types (sedan, SUV, pickup truck) for the two powertrains (ICEV and BEV) that we have used in the present study were compiled from GREET[1,2]. The life cycle data included the GHG emissions from the various stages of the life cycle of the different cars: production of all vehicle components (body, powertrain, transmission, chassis, traction motor, generator, electric control unit), fluids used over the lifetime of the vehicle (e.g., 39 oil changes and 19 windshield fluid refills), vehicle batteries (lead-acid batteries in both powertrains, and Li-ion traction batteries in BEV vehicles), and the assembly and disposal of the vehicle. Vehicle assembly, including battery assembly, takes place in the US. Emissions from electricity used in manufacturing processes reflects the US average grid mix. The vehicle lifetime for all the types of cars for both powertrains was assumed to be 150,000 km, which is a conservative approach. The cumulative impacts from the LCA did not include the recycling or second-life usage of the Li-ion battery, which if investigated could offset a small fraction of the net GHG emissions.

Table 1 below shows the cradle-to-grave emissions inventory except for the use-phase, for the cars used in the present study. Figure 1 represents the breakdown in a stacked bar graph. One can notice the difference in impacts between the two powertrains when the use-phase emissions are excluded.

Car Type	Powertrain	Disposal	Assembly	Batteries	Fluids	Components
Sedan	ICEV	0.191896	0.727146	0.038792	0.763703	4.398661
	BEV	0.191896	0.727146	5.516145	0.183136	3.834258
SUV	ICEV	0.222879	0.727146	0.057649	1.144074	5.429686
	BEV	0.222879	0.727146	7.348491	0.314131	4.819855
Pickup	ICEV	0.280969	0.727146	0.057649	1.144074	6.923653
	BEV	0.280969	0.727146	8.887361	0.314131	6.063048

Table 1Cradle-to-gate GHG emissions (tons of CO2e) (up to manufacturing of cars)

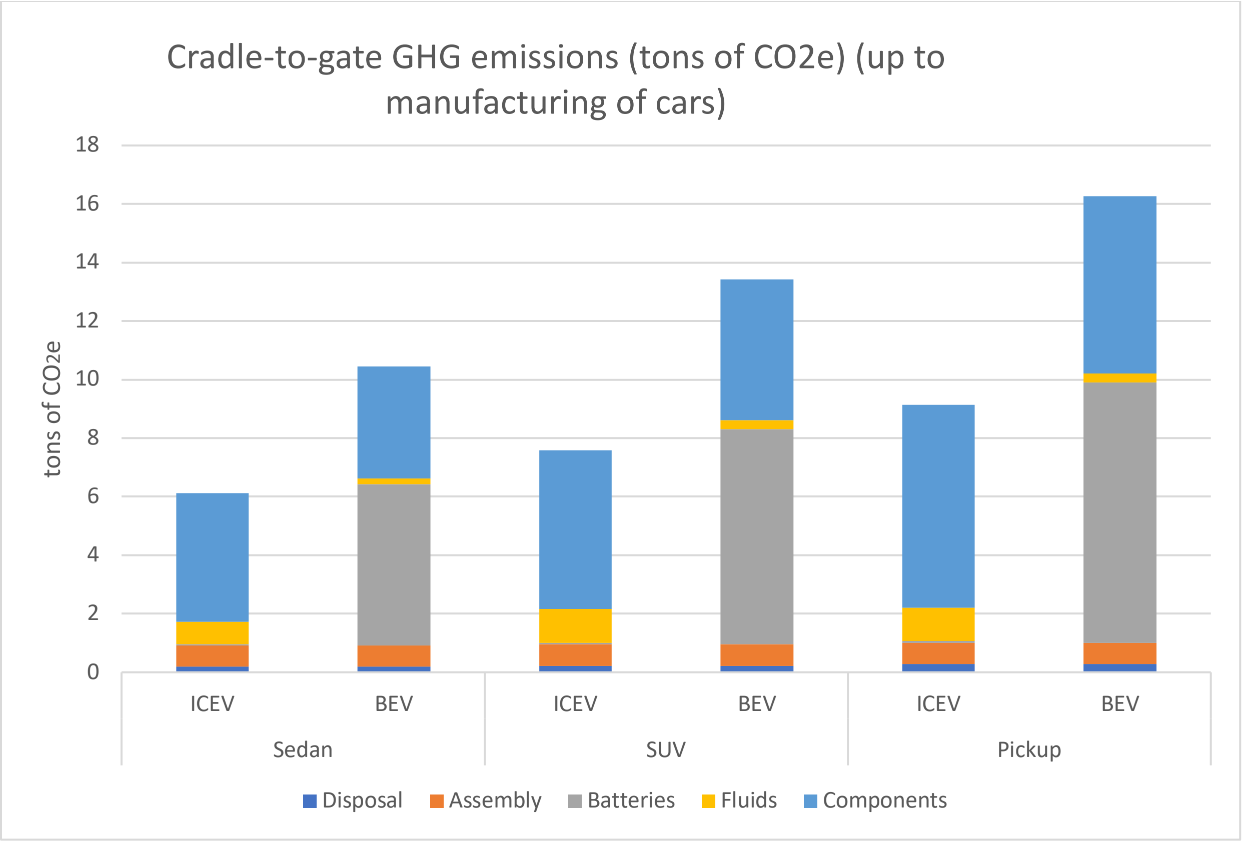

Figure 1 Cradle-to-gate GHG Emissions

Since we have the detailed use-phase data of Georgia Tech's fleet, this was used to model the life cycle GHG impacts during the use-phase of the different types of cars for both the powertrains under consideration.

Figure 2 below gives the trend of the number of miles traveled by the different vehicles in the fleet on an annual basis for the year 2022. We have only represented the top 50 most used vehicles on campus in this figure as the bulk of the analysis focuses on this portion of the fleet, as it accounts for half the life cycle emissions of the fleet. Figure 3 shows the corresponding fuel consumption of these cars over a year period. The differences in fuel consumption can be attributed to the type of car and the specific function of the car in the fleet.

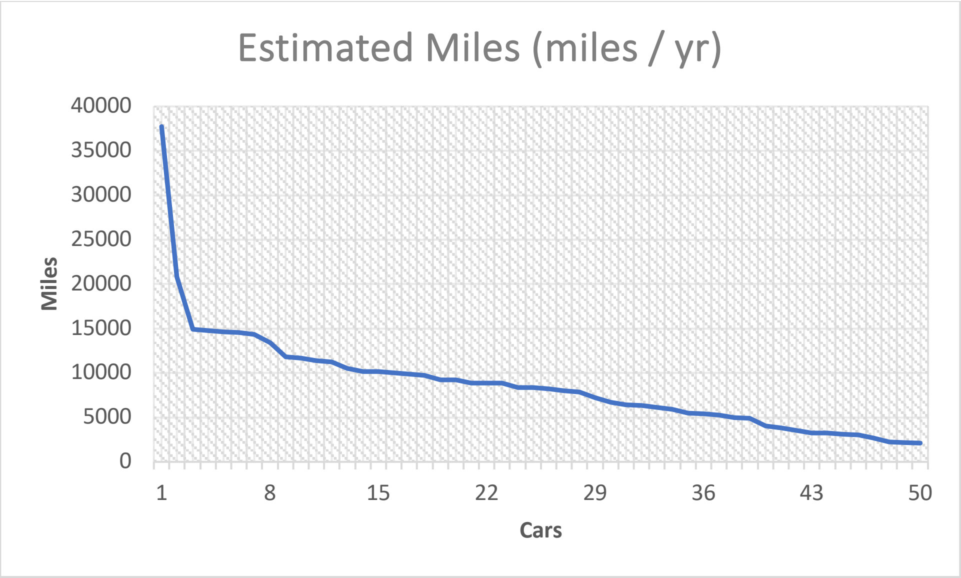

Figure 2 Yearly miles traveled by top 50 most used cars.

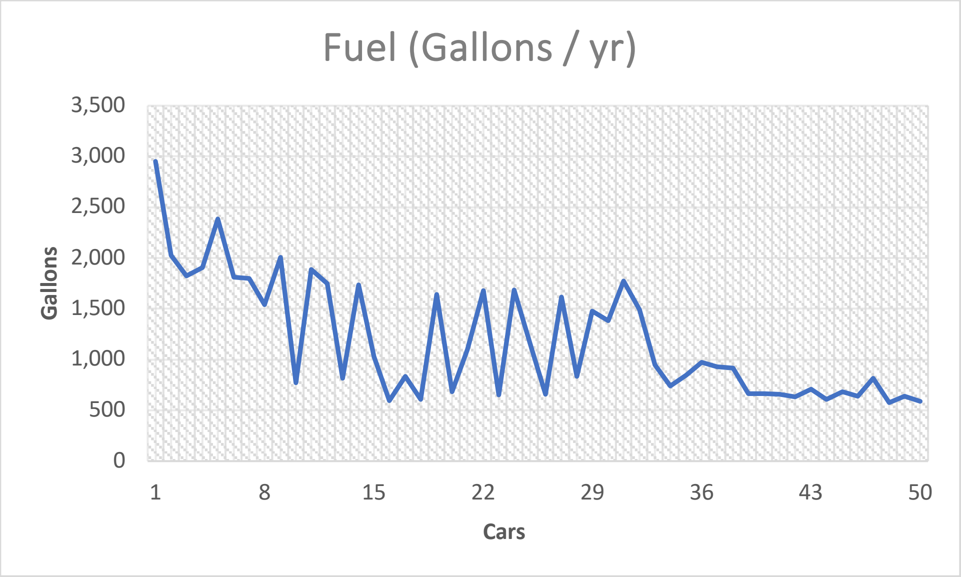

Figure 3 Fuel usage

This data was then used to estimate the annual use-phase emissions of the existing fleet. Figure 4 below shows the annual use-phase GHG emissions of the 50 most used vehicles in the fleet. The values range from roughly 26 tons of CO_2e to 5 tons of CO_2e. One can notice that the annual use-phase emissions are much higher when compared to the cumulative emissions from the other stages of the ICEV life cycle, with a maximum of roughly 9 tons of CO_2e. Figure 5 below shows the life-cycle distribution of emissions across different stages of an ICEV based on the average annual use-phase emissions of the most-used cars in the fleet. The disparity between the impacts of the use-phase compared to the other phases is apparent.

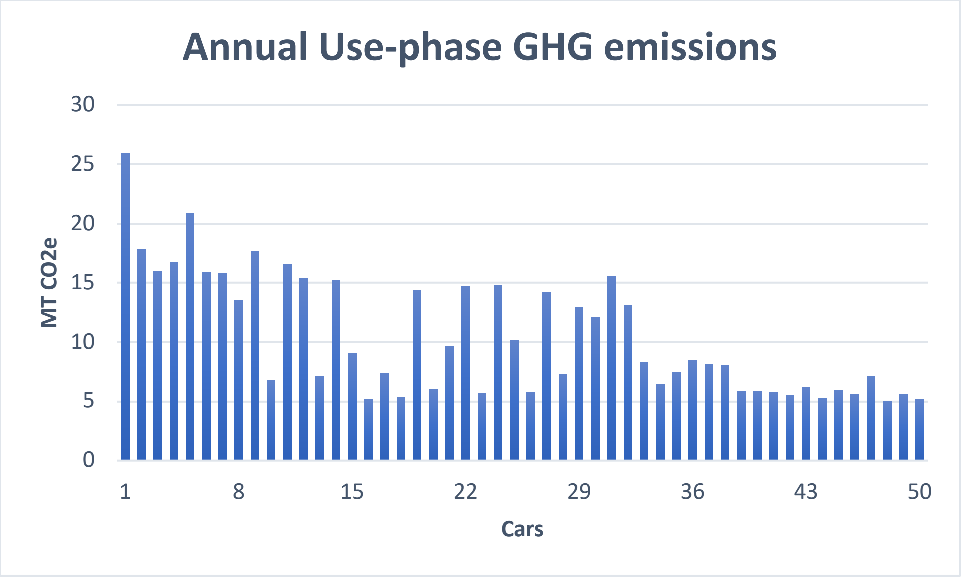

Figure 4 Annual Use-phase GHG emissions

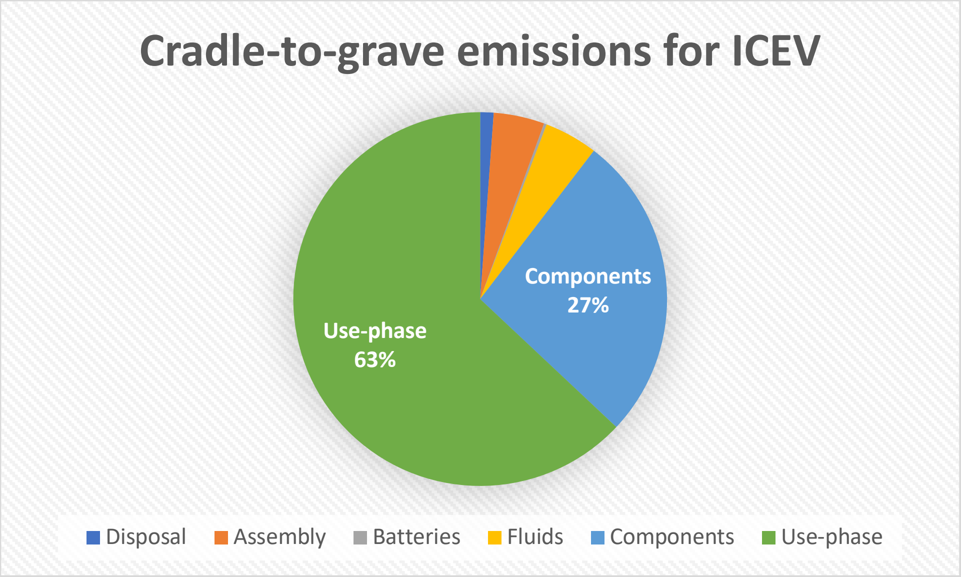

Figure 5 Cradle-to-grave emissions for ICEV

To calculate the impacts of replacing the existing fleet vehicles with electric alternatives, we choose EVs of similar vehicle type (sedan, SUV, truck) based on requirements mentioned in contracts (Georgia Tech being a public university). The compiled list of alternatives for the 50 most used cars was as follows:

Table 2 Electric Alternatives

S. No.	Vehicle Model	Vehicle Replacement
1.	2012 Ford Econoline	E-Transit
2.	2021 Chevrolet Tahoe	Chevrolet Bolt EUV
3.	2019 Ford Transit	E-Transit
4.	2018 Ford Transit	E-Transit
5.	2018 Ford Explorer	Chevrolet Bolt EUV
6.	2019 Ford Transit	E-Transit
7.	2019 Ford Transit	E-Transit
8.	2018 Chevrolet Tahoe	Chevrolet Bolt EUV
9.	2017 Ford Explorer	Chevrolet Bolt EUV
10.	2003 Ford F-150	F-150 Lightning
11.	2021 Ford Explorer	Chevrolet Bolt EUV
12.	2021 Ford Explorer	Chevrolet Bolt EUV
13.	2013 Ford Econoline	E-Transit
14.	2021 Ford Explorer	Chevrolet Bolt EUV
15.	1993 Ford Club Wagon	E-Transit
16.	2008 Toyota Tacoma	F-150 Lightning
17.	2017 Ford F-350 Sd	F-150 Lightning
18.	2017 Ford Expedition	E-Transit
19.	2019 Ford Explorer	Chevrolet Bolt EUV
20.	2013 Ford Econoline	E-Transit
21.	2007 Ford Econoline	E-Transit
22.	2019 Ford Explorer	Chevrolet Bolt EUV
23.	2011 Ford Econoline	E-Transit
24.	2014 Dodge Charger	Mustang Mach-E
25.	2007 Ford Econoline	E-Transit
26.	2021 Ford F-550	F-150 Lightning
27.	2018 Ford Explorer	Chevrolet Bolt EUV
28.	2013 Ford F-150	F-150 Lightning
29.	2017 Ford Explorer	Chevrolet Bolt EUV
30.	2018 Ford Explorer	Chevrolet Bolt EUV
31.	2010 Ford F-150	F-150 Lightning
32.	2015 Dodge Charger	Mustang Mach-E
33.	2017 Ford Explorer	Chevrolet Bolt EUV
34.	2011 Ford Expedition	Chevrolet Bolt EUV
35.	2016 Kia Sedona	Chevrolet Bolt EUV
36.	2018 Ford Taurus	Mustang Mach-E
37.	2016 Ford Transit Connect	E-Transit
38.	2008 Ford Explorer	Chevrolet Bolt EUV
39.	2016 Ford Escape	Chevrolet Bolt EUV
40.	2015 Ford Transit	E-Transit
41.	2015 Dodge Grand Caravan	Chevrolet Bolt EUV

42.	2004 Ford F-450 Sd	F-150 Lightning
43.	2014 Dodge Charger	Mustang Mach-E
44.	2017 Ford Taurus	Mustang Mach-E
45.	2014 Ford F-150	F-150 Lightning
46.	2013 Ford F-350 Sd	F-150 Lightning
47.	2016 Ford F-150	F-150 Lightning
48.	2014 Ford F-150	F-150 Lightning
49.	2016 Ford Explorer	Chevrolet Bolt EUV
50.	2008 Ford Crown Victoria	Chevrolet Bolt

The use-phase emissions of the electrified fleet was then estimated using the average grid carbon intensity of the US state of Georgia and the mileage of the electric alternatives as taken from their respective specifications mentioned on their websites.

Figure 6 shows the estimated annual electricity consumption for the 50 cars and figure 7 shows the annual use-phase GHG emissions for the BEV fleet.

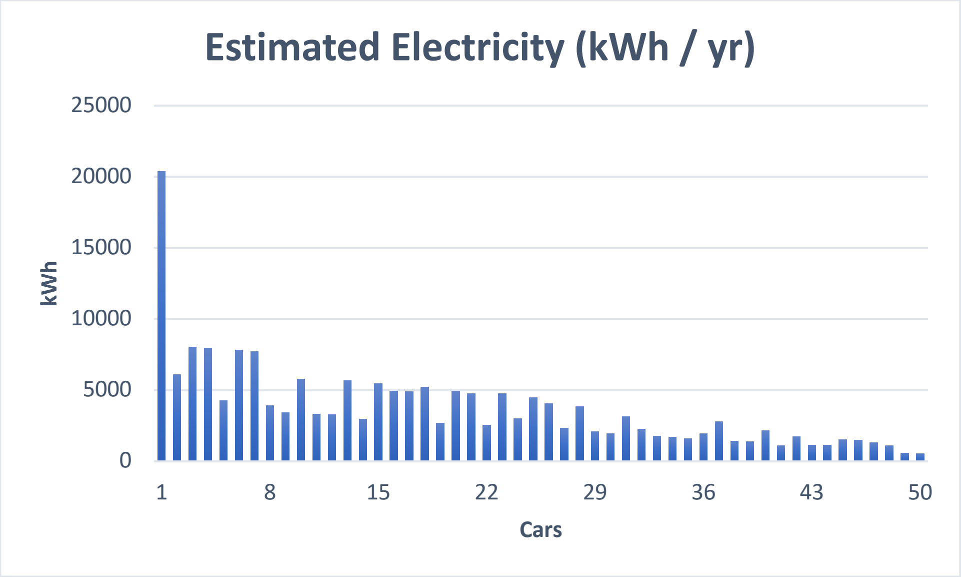

Figure 6

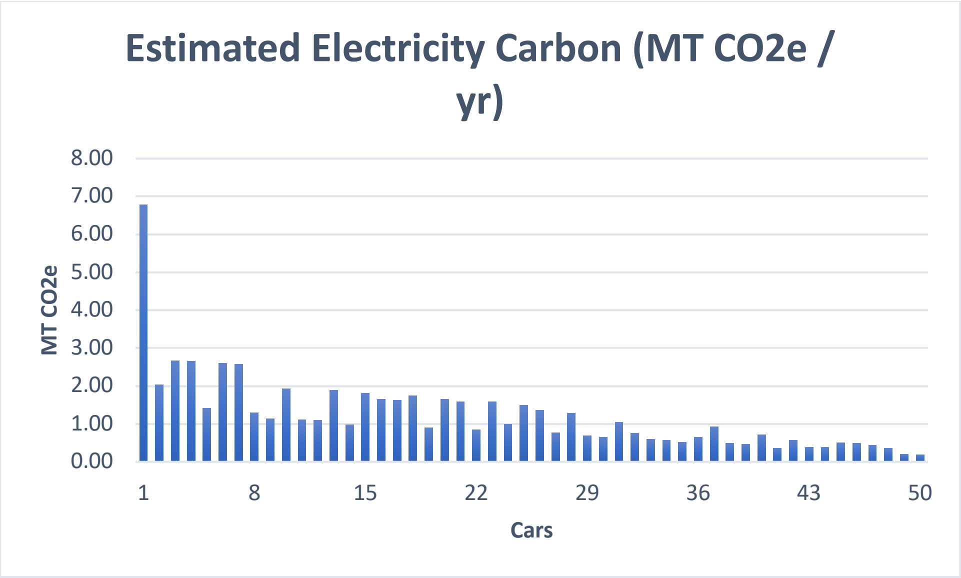

Figure 7

Figure 8 below compares the use-phase emissions of ICEV and BEV for the 50 most used cars in the fleet. It becomes clear that the difference in emissions between the two powertrains is the major source of carbon benefits in the case of EV adoption.

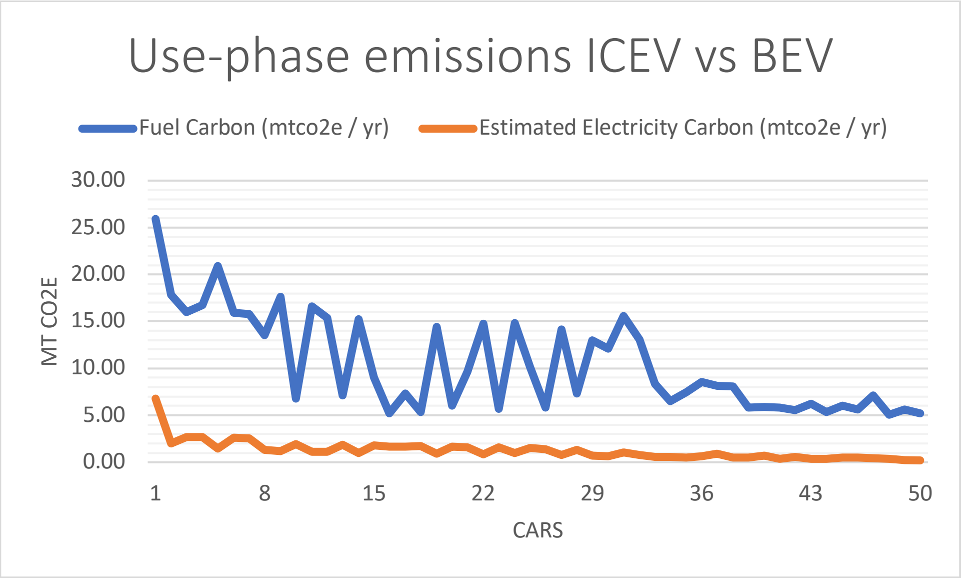

Figure 8

Next, the cumulative cradle-to-grave life cycle GHG emissions for both the ICEV fleet and the proposed BEV fleet were calculated using the data presented above. The GHG emissions were normalized for a period of 1-year based on the miles travelled and the assumed lifespan of 150,000 km for all vehicle types. This was done since we have granular annual use-case data for the year 2022, hence, reducing the need for assumptions on the use-phase of the life cycle. Table 3 below shows the normalized annual life-cycle GHG emissions and the resulting carbon savings.

Table 3 Annual embodied carbon: ICEV vs BEV

**S.No.**	**Total carbon ICEV ($MTCO_2e$)**	**Total embodied carbon (EV) ($MTCO_2e$)**	**Carbon Savings($MTCO_2e$)**
1.	28.9866	12.218	16.8056
2.	21.8723	3.065	18.8155
3.	19.1670	4.367	14.8110
4.	18.4075	2.473	15.9403
5.	17.9170	4.790	13.1414
6.	17.3384	2.392	14.9527
7.	17.2118	4.819	12.4071
8.	17.0686	4.702	12.3804
9.	16.9771	4.640	12.3510
10.	16.2009	2.169	14.0376
11.	16.0981	2.363	13.7407
12.	15.9285	2.134	13.7995
13.	15.4732	2.210	13.2687
14.	15.3264	1.852	13.4789
15.	14.9959	1.939	13.0614
16.	14.7070	1.684	13.0271
17.	14.4184	2.814	11.6118
18.	13.5844	1.670	11.9184
19.	13.4477	1.514	11.9380
20.	12.5673	1.405	11.1658
21.	10.8284	2.695	8.1415
22.	10.3600	2.879	7.4901
23.	9.8631	3.279	6.5939
24.	8.7238	1.291	7.4358
25.	8.5829	1.695	6.8928
26.	8.3894	1.049	7.3428
27.	7.8065	1.145	6.6645
28.	9.4893	3.362	6.1366
29.	8.0896	2.659	5.4380
30.	7.9978	3.415	4.5931
31.	7.3991	0.913	6.4881

32.	7.9017	3.972	3.9405
33.	6.8840	1.244	5.6435
34.	6.4951	0.859	5.6380
35.	6.7743	2.980	3.8033
36.	6.2993	1.055	5.2474
37.	6.1864	1.307	4.8830
38.	6.1666	1.024	5.1449
39.	6.6111	2.796	3.8230
40.	6.0497	0.809	5.2432
41.	6.4282	2.859	3.5774
42.	6.8310	1.021	5.8123
43.	5.7372	0.449	5.2898
44.	5.9060	1.207	4.7021
45.	6.1187	3.145	2.9835
46.	5.5827	0.859	4.7263
47.	6.1841	3.407	2.7857
48.	5.3230	0.433	4.8906
49.	5.2810	0.769	4.5137
50.	8.9610	1.438	7.5263
**Total**	**556.9450**	**109.019**	**436.0443**

Once the carbon savings were computed, the carbon payback was calculated as a ratio of the difference in the GHG impacts of manufacture for the two car classes (ICEV and BEV) to the difference in annual use-phase emissions. Figure 9 below shows the carbon payback period of the 50 most used cars in the fleet, with the payback ranging from as little as 3 months to 24 months. This means that replacing the most used car in the fleet with an electric alternative would offset the life-cycle emissions of the car in under 3 months.

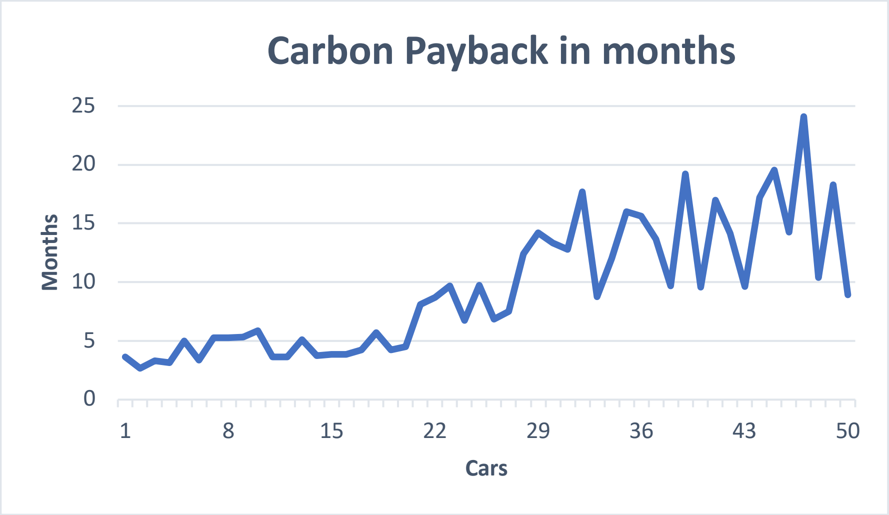

Figure 9

Now that we have the carbon savings from fleet electrification for Georgia Tech using granular annual use-case data, we estimated the carbon savings and carbon paybacks in different states of the US by assuming that Georgia Tech was an archetypical university in each of these states. This analysis helped us to understand the dynamics of fleet electrification in 'dirtier' grids and the carbon benefits/demerits of the same. Table 4 below shows the state-wise grid carbon intensity in the US.

Table 4 State wise Grid Carbon intensity[3]

State	Grid carbon intensity (lb/MWh)
WV	1,932
KY	1,738
WY	1,733
MO	1,673
IN	1,600
UT	1,497
HI	1,369
ND	1,350
OH	1,195

NE	1,177
CO	1,176
WI	1,172
MT	1,063
NM	1,063
AR	1,046
AK	1,011
MI	1,002
DE	970
KS	875
TX	847
LA	846
FL	840
RI	829
MS	822
IA	773
MN	767
MA	757
GA	734
OK	722
AL	720
PA	715
NV	704
MD	674
AZ	672
IL	670
NC	663
TN	657
VA	600
SC	530
CT	519
NJ	463
NY	452
CA	391
SD	285
OR	282
NH	278
ME	261
ID	238
WA	199
VT	1

These grid intensity factors were then incorporated into the LCA of the EV fleet to arrive at the GHG impacts due to the representative fleet in different states. Table 5 below shows the cumulative GHG emissions for a completely electric fleet in each of these states. The column titled instant electrification gives the GHG emissions from total electrification while the “Targeted Electrification” column gives the GHG emissions from electrification of the top 50 used cars. The carbon payback in months is the average payback period of the top 50 most used cars in the representative fleet. The key takeaway from this result was the carbon payback period of under 17 months for the most used cars in the fleet even with the ‘dirtiest’ grid in the US. This further highlights the vast difference in the life cycle GHG impacts of ICEVs when compared to BEVs, even when the use-phase electricity is consumed from a relatively dirty electric grid. Table 6 shows a similar analysis but with an international perspective.

Table 5 Lifecycle GHG results of Fleet Electrification by state.

State	Total carbon (instant electrification)	Targeted electrification	Carbon payback (months)
WV	500.528	222.089	16.77
KY	463.847	205.757	14.13
WY	462.902	205.336	14.08
MO	451.557	200.285	13.54
IN	437.754	194.140	12.97
UT	418.279	185.469	12.29
HI	394.077	174.694	11.61
ND	390.485	173.094	11.51
OH	361.178	160.046	10.85
NE	357.774	158.530	10.78
CO	357.585	158.446	10.78
WI	356.829	158.110	10.76
MT	336.219	148.934	10.38
NM	336.219	148.934	10.38
AR	333.005	147.502	10.32
AK	326.387	144.556	10.21
MI	324.686	143.798	10.18
DE	318.635	141.105	10.08
KS	300.673	133.107	9.81
TX	295.378	130.750	9.73
LA	295.189	130.666	9.73
FL	294.055	130.161	9.71
RI	291.975	129.235	9.68
MS	290.652	128.645	9.66
IA	281.387	124.520	9.54
MN	280.252	124.015	9.52
MA	278.361	123.173	9.5

GA	274.013	121.237	9.44
OK	271.744	120.227	9.41
AL	271.366	120.059	9.4
PA	270.420	119.638	9.39
NV	268.340	118.712	9.37
MD	262.668	116.186	9.3
AZ	262.290	116.018	9.29
IL	261.912	115.850	9.28
NC	260.588	115.260	9.27
TN	259.454	114.755	9.26
VA	248.676	109.957	9.13
SC	235.441	104.064	8.98
CT	233.361	103.138	8.95
NJ	222.773	98.424	8.84
NY	220.693	97.498	8.82
CA	209.159	92.362	8.7
SD	189.117	83.439	8.5
OR	188.549	83.186	8.49
NH	187.793	82.850	8.49
ME	184.579	81.419	8.46
ID	180.230	79.482	8.42
WA	172.856	76.199	8.35
VT	135.418	59.531	8.03

Table 6 Lifecycle GHG results of Fleet Electrification by Country.

**Country**	**Grid carbon intensity(g CO2/kWh)**	**Total carbon (instant electrification)**	**Targeted electrification**	**Carbon payback**
Australia	503	344.903	152.800	10.53
Belgium	165	204.009	90.069	8.64
Brazil	102	177.748	78.377	8.39
Canada	128	188.586	83.203	8.49
China	531	356.575	157.996	10.76
Denmark	181	210.679	93.039	8.71
Finland	131	189.836	83.759	8.51
France	85	170.661	75.222	8.33
Germany	385	295.715	130.900	9.74
Hong Kong	684	420.352	186.392	12.36
India	632	398.676	176.741	11.73
Italy	372	290.296	128.487	9.66
Netherlands	355	283.210	125.332	9.56
Norway	26	146.067	64.272	8.12
Russia	367	288.212	127.559	9.63
Saudi Arabia	571	373.248	165.420	11.11
South Africa	709	430.773	191.032	12.71
Spain	217	225.685	99.720	8.87
Sweden	45	153.987	67.798	8.19
Switzerland	46	154.404	67.984	8.19
United Kingdom	257	242.359	107.144	9.05
United States	367	288.212	127.559	9.63

3. Evolution of Grid Mixes Over Time

Figure 10 below shows the breakdown of the world's electricity profile over time. One can notice that there has been an upward trend in the percentage of renewables over the last twenty years or so, but it is important to also look at the absolute amount of electricity being produced over this same period and not just the breakdown of it. Figure 11 below shows this latter trend over the same period of time, and one can notice how the demand for electricity has increased over two-fold in the past thirty years.[4] The graph shows the increase in electricity generation from fossil fuel sources (coal, oil, and gas) to keep up with this increased demand along with the increase in generation through renewables (solar, wind, bioenergy, and nuclear). The trends of a few select countries are also included to highlight regional differences in grid evolution over time. One can notice that the economic status of the country, the availability of natural resources (both fossil and renewable), and the rate of increase in power demand are key factors that shape the evolution of grid mixes.

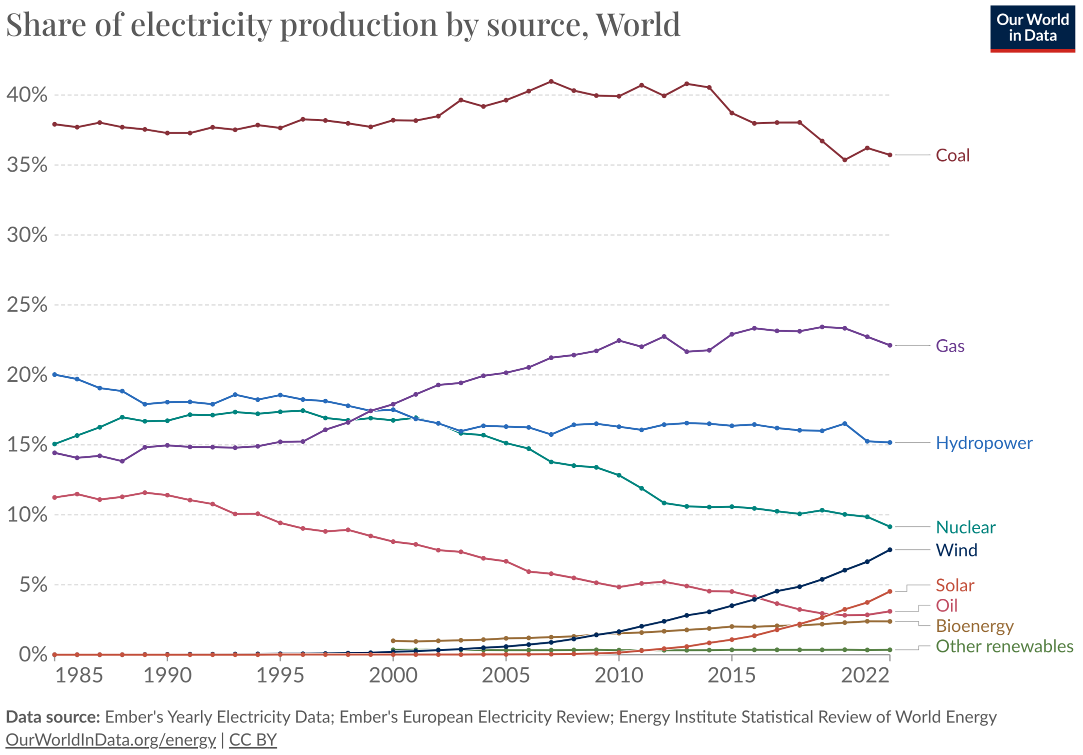

Figure 10 World Electricity mix over time.

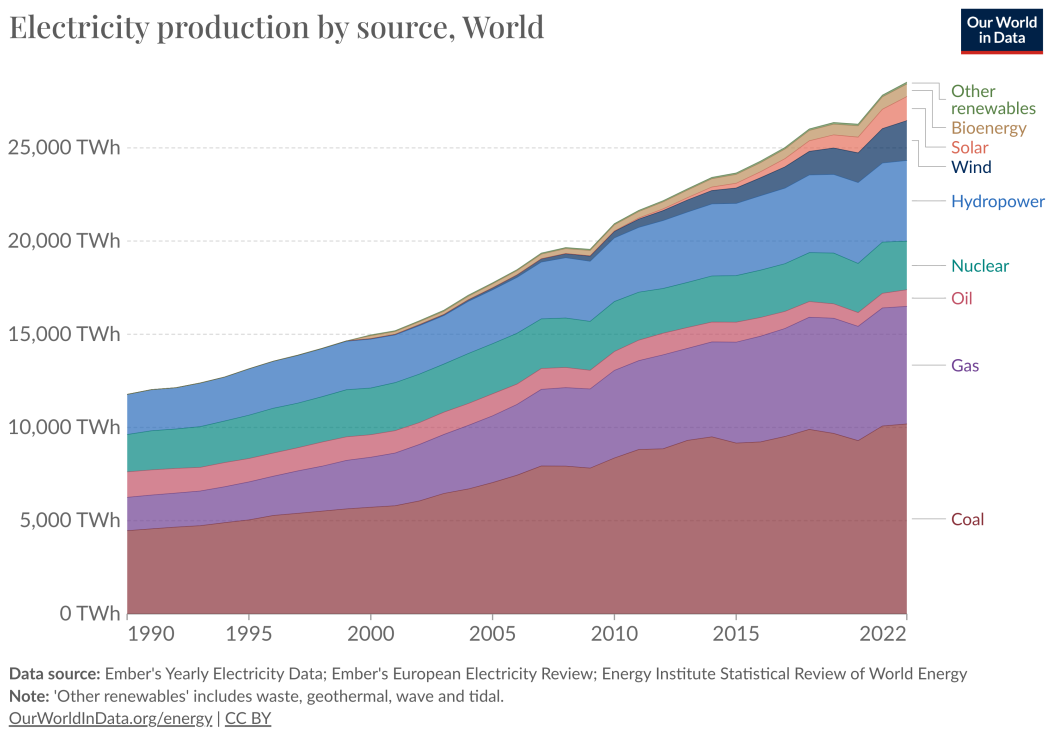

Figure 11 World Electricity production by source

Other data and visualizations as a supplement

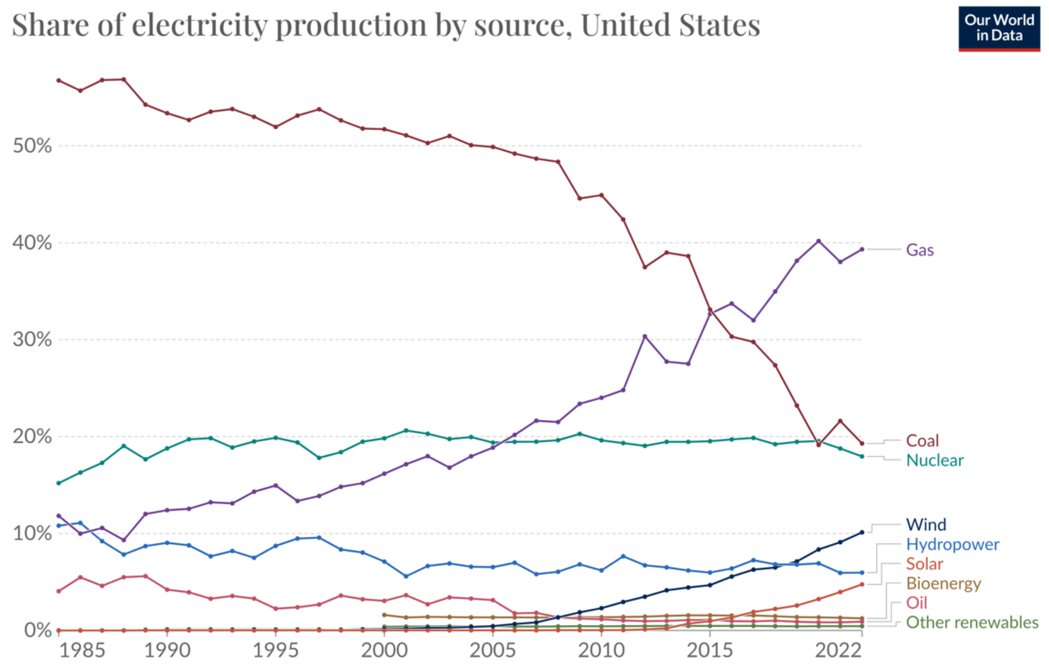

Data source: Ember's Yearly Electricity Data; Ember's European Electricity Review; Energy Institute Statistical Review of World Energy
OurWorldInData.org/energy | CC BY

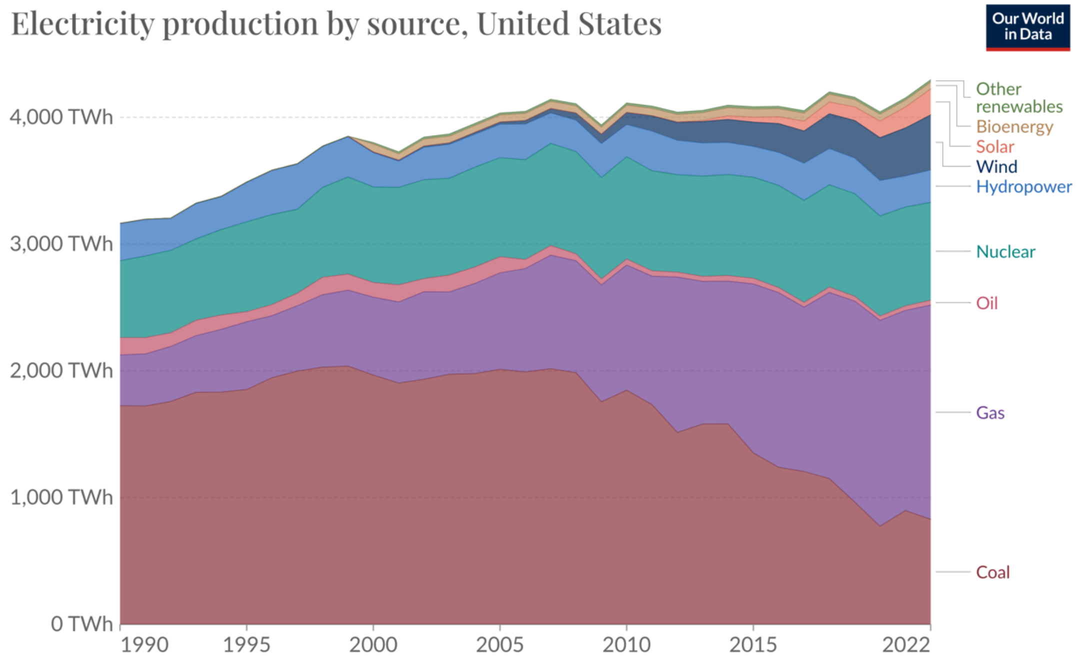

Data source: Ember's Yearly Electricity Data; Ember's European Electricity Review; Energy Institute Statistical Review of World Energy
Note: 'Other renewables' includes waste, geothermal, wave and tidal.
OurWorldInData.org/energy | CC BY

Share of electricity production by source, India

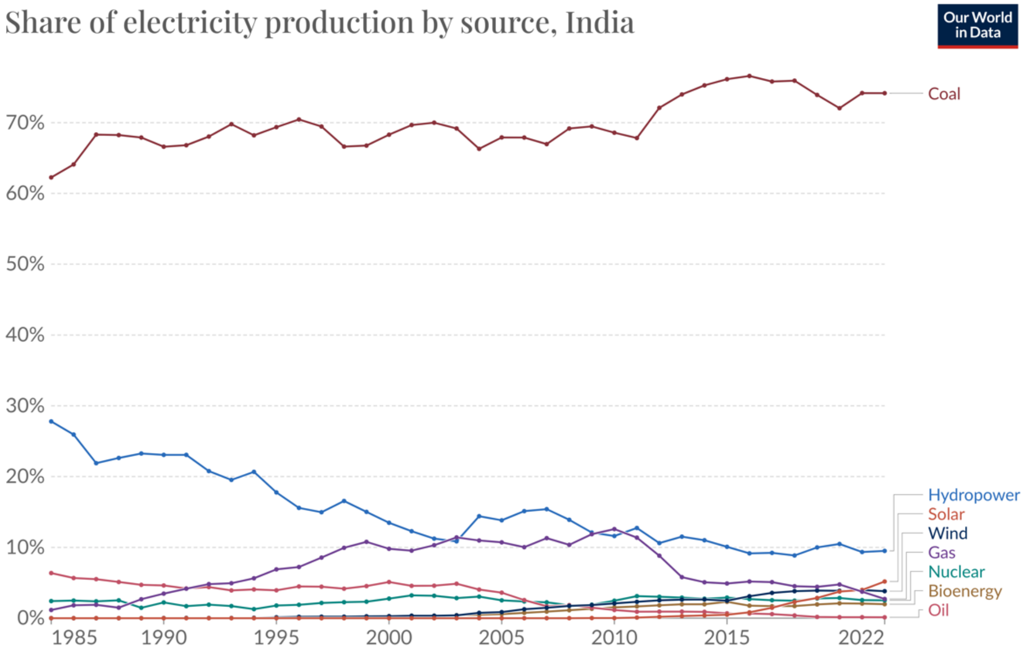

Data source: Ember's Yearly Electricity Data; Ember's European Electricity Review; Energy Institute Statistical Review of World Energy
OurWorldInData.org/energy | CC BY

Electricity production by source, India

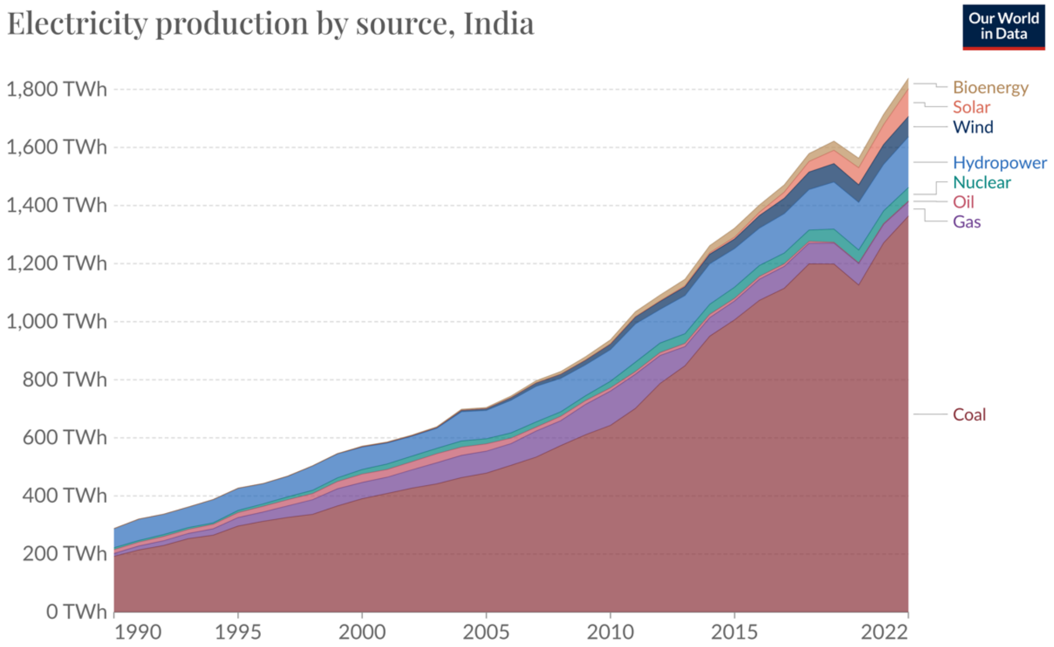

Data source: Ember's Yearly Electricity Data; Ember's European Electricity Review; Energy Institute Statistical Review of World Energy
Note: 'Other renewables' includes waste, geothermal, wave and tidal.
OurWorldInData.org/energy | CC BY

Share of electricity production by source, Sweden

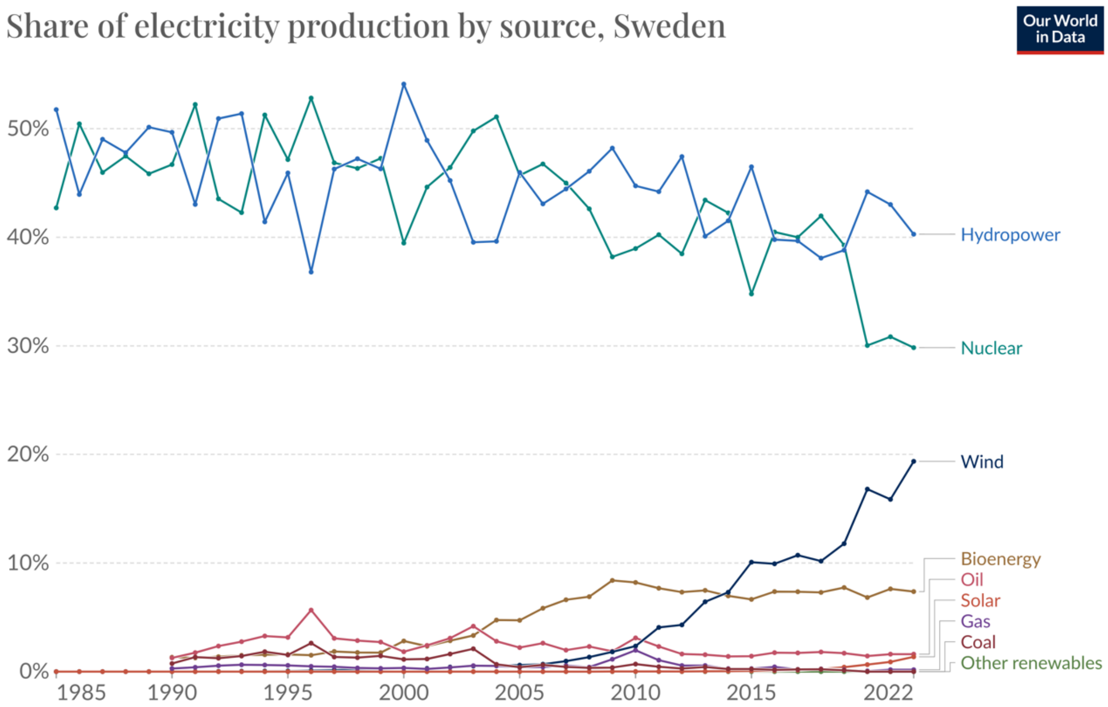

Data source: Ember's Yearly Electricity Data; Ember's European Electricity Review; Energy Institute Statistical Review of World Energy
OurWorldInData.org/energy | CC BY

Electricity production by source, Sweden

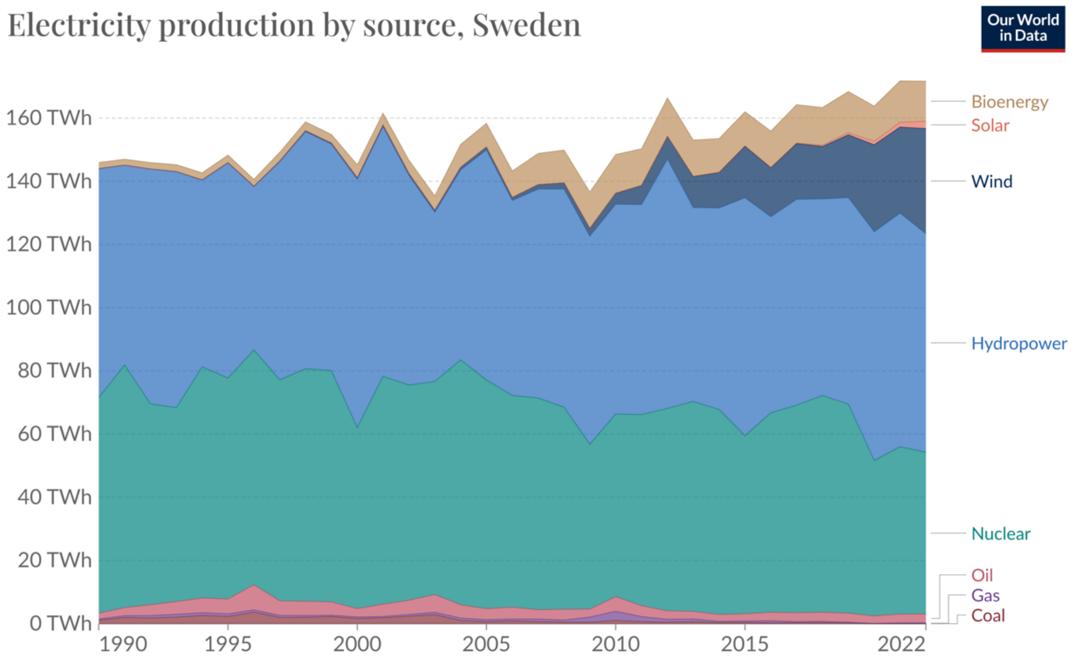

Data source: Ember's Yearly Electricity Data; Ember's European Electricity Review; Energy Institute Statistical Review of World Energy
Note: 'Other renewables' includes waste, geothermal, wave and tidal.
OurWorldInData.org/energy | CC BY

References:

1. Wang, M. *et al. Summary of Expansions and Updates in GREET® 2021*. https://www.osti.gov/biblio/1824336 (2021) doi:10.2172/1824336.
2. Woody, M. *et al.* The role of pickup truck electrification in the decarbonization of light-duty vehicles. *Environ. Res. Lett.* **17**, 034031 (2022).
3. U.S. power sector carbon index by state 2022. *Statista* https://www.statista.com/statistics/1133295/electric-sector-carbon-dioxide-emission-rate-by-state-united-states/.
4. Ritchie, H., Rosado, P. & Roser, M. Electricity Mix. *Our World in Data* (2023).
